# Supplementary material for: Arthropod species loss underpins biomass declines
Source: Nat Ecol Evol. 2025 Dec 2;10(1):83–94. doi: 10.1038/s41559-025-02909-y (PMC12789023; doi:10.1038/s41559-025-02909-y)
Supplement: Supplementary file 1 — Supplementary Methods, Notes 1–4, Tables 1–26 and Figs. 1–11. [file 41559_2025_2909_MOESM1_ESM.pdf]

nature ecology & evolution

Supplementary information https://doi.org/10.1038/s41559-025-02909-y

Arthropod species loss underpins biomass declines

In the format provided by the authors and unedited

Table of Contents

Supplementary Methods 1

The ecological Price Equation 1

Supplementary Note 1: Alternative modelling approaches 2

Supplementary Note 2: Sensitivity analyses of the control 3

Supplementary Note 3: Presence-absence analyses 4

Supplementary Note 4: Sensitivity analyses of species with low sampling probabilities 4

Supplementary Tables 6

Supplementary Figures 37

Supplementary References 48

Supplementary Methods

The ecological Price Equation

The 5-part ecological Price equation partitions changes in ecosystem function, or in our case biomass (BM), between two communities into five components: Species richness loss and gain (SRE.L, SRE.G), species identity loss and gain (SIE.L, SIE.G) and context-dependent effects (CDE), which in our case is attributable to abundance changes of persisting species (ABU). These definitions follow ref. [1], except for renaming the fifth component from CDE to ABU. The following equations are modified after ref. [1]:

$$\Delta BM = SRE.L + SRE.G + SIE.L + SIE.G + CDE$$

with (see Supplementary Table 1 for the definitions of the variables):

$$SRE.L = (s_c - s) \cdot \bar{z}$$

$$SRE.G = (s' - s_c) \cdot \bar{z}'$$

$$SIE.L = s_c \cdot (\bar{z}_c - \bar{z})$$

$$SIE.G = -s_c \cdot (\bar{z}'_c - \bar{z}')$$

$$ABU = s_c \cdot (\bar{z}'_c - \bar{z}_c)$$

Supplementary Table 1 Definitions of variables used in the ecological 5-part Price equation modified after ref. [1]

Variable	Definition	Equation
z, z′	Contribution of an individual species to biomass in the baseline (or comparison) community	
s, s′	Species richness of baseline (or comparison) community	
s_c	Number of shared species between the baseline and comparison community	
$\bar{z}, \bar{z}'$	Mean biomass of species in the baseline (or comparison) community	$\bar{z} = \frac{1}{s}\sum_{i \in X} z_i$ $\bar{z}' = \frac{1}{s'}\sum_{j \in X'} z'_j$
$\bar{z}_c, \bar{z}'_c$	Mean biomass of only the shared species, in either the baseline or comparison community	$\bar{z}_c = \frac{1}{s_c}\sum_{k \in X \cap X'} z_k$ $\bar{z}'_c = \frac{1}{s_c}\sum_{k \in X \cap X'} z'_k$

Supplementary Note 1: Alternative modelling approaches

Temporal patterns of biomass change components obtained from restricted moving average comparisons (discussed in the main body of the paper) were largely the same as when analyzed with fixed baseline comparisons and moving average comparisons (Extended Data Fig. 4, 5, Supplementary Fig. 11). However, in the Biodiversity Exploratories the moving average analysis estimated an increase of gained biomass associated with species richness gains over time, while no other analysis did (Extended Data Fig. 5b). This reflects the weakness of unrestricted moving average analyses: effects within each time window are averaged over the whole study period, leading to confounding of systematic effects. For example, as species richness is declining over time, estimated gains are higher in early years than in late years; small moving windows of e.g. 1 year, however, will average the high early gains and the low late

gains, leading to the underestimation of species richness gains in early years and overestimation of species richness gains in late years. Therefore, we consider restricting the moving windows to comparisons including any of the first five years as more realistic, reflected by the consistency of trends obtained by restricted moving average comparisons and fixed baseline comparisons, i.e. the original time series. In the Biodiversity Exploratories, model intercepts and predicted absolute values of biomass losses were much more extreme in the fixed baseline comparisons than in the restricted and unrestricted moving average analyses (Extended Data Fig. 5). This reflects the weakness of fixed baseline analyses: all comparisons rely on the value of one single baseline (2008), which, in this case, was an exceptionally good year for arthropod abundances[2]. Therefore, relative losses in the following years were much higher (especially for abundance declines, Supplementary Fig. 11) than in moving average analyses, where multiple baseline years were included. As discussed in the main manuscript, we conclude that restricted moving average comparisons yield more robust and generalizable trends.

Supplementary Note 2: Sensitivity analyses of the control

When removing the control (within-year, between-replicates turnover of the baseline; year 0) no significant relationships of time and biomass change associated with species richness loss, species identity and abundance change was detected (Supplementary Fig. 1, 2). While this may partly reflect reduced statistical power due to our subsampling of available datapoints (see methods), it highlights the importance of the reference for expected turnover in year 0, that is turnover due to detection “noise”. Otherwise, temporal turnover cannot be distinguished from turnover due to detection probabilities, which is a general issue of the Price equation[1,3]. In fact, excluding a baseline from a time series of relative change generally biases the whole relationship: For example, the sensitivity analysis showed that none of the modelling approaches detected an overall biomass decline in the Biodiversity Exploratories, but rather slight increases (Supplementary Fig. 2f). While this supports findings of fluctuating arthropod

numbers in recent years[4], we argue that change always needs to be evaluated relative to the baseline, that includes 2008 in this case (which had the highest arthropod abundance, species richness and biomass, see above). The baseline for relative total biomass change naturally is set to 0, with sharp drops in the first years compared to 2008 and slight recovery in later years in the Biodiversity Exploratories. Excluding the baseline (0) consequently sets the state of sharp decline as a new baseline, biasing the relationship towards positive estimates. As for the fact that 2008 had the highest arthropod abundance, diversity and biomass, there is no indication for an abiotic, management or sampling anomaly, but rather reason to assume that it reflects on a real trend and should be included in the analyses (see ref [2]).

Supplementary Note 3: Presence-absence analyses

Looking at presence-absence data only, that is the individual biomass per species, lost and gained species initially had above-average biomass relative to their respective communities. Mirrored to the analyses including species abundances in the main manuscript, this effect also stagnated or declined towards the communities' average value over time, while biomass loss associated with species richness loss increased (Extended Data Fig. 6 - 9; Supplementary Tables 6, 7). Mirrored patterns between species identity components based on individual species biomass (above-average members are lost and gained) and the main analysis including abundance (below-average members are lost and gained, i.e. rare species) were expected and reinforce our findings, as the largest species in natural communities usually are rare[5].

Supplementary Note 4: Sensitivity analyses of species with low sampling probabilities

Excluding all species from the analyses that occurred in less than 10% or 30% of all plots per year per research program did not change temporal patterns, but reduced the biomass change associated with species richness turnover, increasing the relative contributions of abundance

change in both research programs (Supplementary Fig. 6, 7, see discussion in the main manuscript). Notably, when removing all species that occurred in less than 30% of all plots per year, gained species had above-average total biomass in later years in the Biodiversity Exploratories (Supplementary Fig. 7d). This may indicate that abundant generalist species are on the rise, shifting abundance patterns in communities towards their numerical dominance [6].

Supplementary Tables

Supplementary Table 2 Summary of the sampled taxa and their abundance and species richness in the Jena Experiment.

Order	Abundance/ Order	Species richness/ Order	Family	Abundance/ Family	Species richness/ Family
Araneae	1752	70	Anyphaenidae	3	1
			Araneidae	134	5
			Clubionidae	1	1
			Dictynidae	17	2
			Gnaphosidae	1	1
			Hahniidae	8	1
			Linyphiidae	755	29
			Lycosidae	44	6
			Philodromidae	3	2
			Phrurolithidae	8	1
			Pisauridae	2	1
			Salticidae	7	3
			Tetragnathidae	478	5
			Theridiidae	275	6
			Thomisidae	16	6
Coleoptera	16086	249	Apionidae	967	22
			Attelabidae	40	1
			Brentidae	11	6
			Buprestidae	2	1
			Byrrhidae	14	2
			Cantharidae	45	4
			Carabidae	79	16
			Cetoniidae	1	1
			Chrysomelidae	11202	36
			Coccinellidae	108	11
			Corylophidae	1	1
			Cryptophagidae	32	3
			Curculionidae	1383	63
			Drilidae	1	1
			Elateridae	34	5
			Helophoridae	4	1
			Hydrophilidae	2	2
			Latridiidae	273	5
			Leiodidae	1	1
			Malachiidae	6	2
			Meloidae	5	1
			Melyridae	4	2
			Monotomidae	1	1
			Mordellidae	5	4
			Nitidulidae	1740	11
			Oedemeridae	17	4

Order	Abundance/ Order	Species richness/ Order	Family	Abundance/ Family	Species richness/ Family
			Phalacridae	9	4
			Ptiliidae	1	1
			Scraptiidae	1	1
			Staphylinidae	97	36
Hemiptera	11162	145	Alydidae	9	1
			Anthocoridae	94	3
			Aphrophoridae	36	4
			Berytidae	5	2
			Cercopidae	3	1
			Cicadellidae	5090	45
			Coreidae	14	3
			Delphacidae	3509	12
			Dictyopharidae	39	1
			Heterogastridae	1	1
			Membracidae	19	2
			Miridae	2033	36
			Nabidae	85	3
			Oxycarenidae	1	1
			Pentatomidae	47	9
			Piesmatidae	1	1
			Pyrrhocoridae	1	1
			Rhopalidae	63	6
			Rhyparochromidae	29	6
			Scutelleridae	2	1
			Tettigometridae	2	1
			Tingidae	79	5
Hymenoptera	10965	135	Andrenidae	19	8
			Aphelinidae	101	1
			Apidae	203	13
			Bethylidae	5	1
			Braconidae	3098	14
			Ceraphronidae	371	1
			Colletidae	5	4
			Crabronidae	27	10
			Cynipidae	131	1
			Diapriidae	272	1
			Dryinidae	9	2
			Elasmidae	3	1
			Embolemidae	1	1
			Encyrtidae	261	1
			Eucoilidae	176	1
			Eulophidae	1527	2
			Eumenidae	3	3
			Eupelmidae	11	1
			Eurytomidae	86	1
			Figitidae	3	1

Order	Abundance/ Order	Species richness/ Order	Family	Abundance/ Family	Species richness/ Family
			Gasteruptiidae	1	1
			Halictidae	112	20
			Ichneumonidae	738	23
			Megaspilidae	86	1
			Melittidae	1	1
			Mymaridae	576	1
			Ormyridae	1	1
			Perilampidae	14	1
			Platygastridae	589	2
			Pompilidae	7	4
			Proctotrupidae	19	1
			Pteromalidae	1695	1
			Scelionidae	661	1
			Tenthredinidae	14	3
			Tetracampidae	63	1
			Tiphiidae	3	2
			Torymidae	69	1
			Trichogrammatidae	3	1
			Vespidae	1	1

Supplementary Table 3 Summary of the sampled taxa and their abundance and species richness in the Biodiversity Exploratories.

Order	Abundance/ Order	Species richness/ Order	Family	Abundance/ Family	Species richness/ Family
Araneae	6666	173	Araneidae	1090	24
			Clubionidae	11	5
			Dictynidae	31	5
			Eutichuridae	2	2
			Linyphiidae	3110	63
			Lycosidae	86	10
			Mimetidae	1	1
			Philodromidae	96	8
			Pisauridae	2	1
			Salticidae	23	5
			Tetragnathidae	409	9
			Theridiidae	1428	24
			Thomisidae	377	16
Coleoptera	38952	728	Anobiidae	2	2
			Anthicidae	22	4
			Apionidae	3884	52
			Bruchidae	80	10
			Buprestidae	1	1
			Byrrhidae	3	2
			Byturidae	1	1
			Cantharidae	1979	26
			Carabidae	180	25
			Cerambycidae	46	12
			Cerylonidae	1	1
			Cholevidae	26	6
			Chrysomelidae	11939	117
			Coccinellidae	2443	31
			Corylophidae	2	2
			Cryptophagidae	50	13
			Curculionidae	6757	128
			Dascillidae	1	1
			Drilidae	3	1
			Elateridae	1184	31
			Hydrophilidae	86	16
			Kateritidae	207	6
			Lagriidae	1	1
			Latridiidae	1301	15
			Leiodidae	5	3
			Lucanidae	1	1
			Malachidae	412	10
			Melyridae	81	5
			Mordellidae	62	9
			Nitidulidae	6521	22
			Oedemeridae	199	9

Order	Abundance/ Order	Species richness/ Order	Family	Abundance/ Family	Species richness/ Family
			Omalisidae	11	1
			Phalacridae	653	13
			Pselaphidae	4	3
			Ptiliidae	5	1
			Pyrochroidae	1	1
			Rhizophagidae	2	2
			Rhynchitidae	1	1
			Salpingidae	1	1
			Scarabaeidae	343	20
			Scirtidae	18	6
			Scolytidae	4	4
			Scraptiidae	11	4
			Silphidae	2	1
			Sphindidae	1	1
			Staphylinidae	410	105
			Throscidae	5	1
Hemiptera	150455	370	Acanthosomatidae	1	1
			Alydidae	7	1
			Anthocoridae	116	7
			Aphrophoridae	4108	8
			Berytidae	101	5
			Cercopidae	17	1
			Cicadellidae	82573	103
			Cixiidae	12	2
			Coreidae	54	5
			Delphacidae	12774	44
			Dictyopharidae	19	1
			Lygaeidae	434	29
			Membracidae	1	1
			Miridae	48162	101
			Nabidae	677	8
			Pentatomidae	715	18
			Piesmatidae	7	2
			Plataspididae	6	1
			Reduviidae	2	1
			Rhopalidae	270	10
			Saldidae	16	3
			Scutelleridae	160	2
			Tettigometridae	14	2
			Tingidae	209	14
Orthoptera	3652	28	Acrididae	3445	14
			Conocephalidae	46	2
			Meconematidae	1	1
			Phaneropteridae	10	3
			Tetrigidae	114	4
			Tettigoniidae	36	4

Supplementary Table 4 Mean arthropod biomass and species richness per sample (replicate) in the baseline years of the restricted moving average analysis (Jena Experiment: 2010, 2012, 2014, Biodiversity Exploratories: 2008-2012), and the median model predictions with 95% confidence intervals (CI) for absolute biomass and species richness loss after 7 years.

**Dataset**	**Mean biomass baseline [mg]**	**Predicted biomass loss after 7 years [mg]**	**Mean species richness baseline [mg]**	**Predicted species loss after 7 years [mg]**
Jena Experiment	192.143	-68.227 (95% CI: -36.168, -99.684)	19.651	-6.458 (95% CI: -3.754, -9.145
Biodiversity Exploratories	677.99	-23.901 (95% CI: -6.806, -51.062)	17.344	-0.21 (95% CI: -0.041, -0.493)

Supplementary Table 5 Median estimates, 95% confidence intervals (2.5th–97.5th percentiles; CI low and CI high), two-sided p-values and median R^2 values across 1000 subsamples without replacement (see methods) of the restricted moving average analysis of overall arthropod responses (square root transformed) in the Jena Experiment. PSR = plant species richness, m/c = marginal/conditional.

Parameter	Estimate (median)	CI low	CI high	p-value	Response	m/c R^2
(Intercept)	-10.717	-11.063	-10.399	<0.001	Species richness loss	0.139/ 0.309
scale(Years passed):	-0.977	-1.467	-0.451	<0.001	Species richness loss	
scale(PSR)	-1.29	-1.69	-0.903	<0.001	Species richness loss	
scale(Years):scale(PSR)	-0.151	-0.723	0.408	0.614	Species richness loss	
(Intercept)	7.758	7.356	8.151	<0.001	Species richness gain	0.119/ 0.361
scale(Years passed):	-0.784	-1.325	-0.05	0.04	Species richness gain	
scale(PSR)	0.979	0.594	1.405	<0.001	Species richness gain	
scale(Years):scale(PSR)	-0.064	-0.548	0.349	0.736	Species richness gain	
(Intercept)	0.969	0.447	1.531	<0.001	Species identity loss	0.047/ 0.146
scale(Years passed):	-0.272	-0.946	0.468	0.444	Species identity loss	
scale(PSR)	1.02	0.363	1.621	0.002	Species identity loss	
scale(Years):scale(PSR)	0.066	-0.72	0.884	0.876	Species identity loss	
(Intercept)	-0.711	-1.263	-0.203	0.008	Species identity gain	0.042/ 0.117
scale(Years passed):	0.576	-0.02	1.155	0.056	Species identity gain	
scale(PSR)	-0.534	-1.106	0.068	0.084	Species identity gain	
scale(Years):scale(PSR)	0.363	-0.422	0.998	0.342	Species identity gain	
(Intercept)	-1.094	-1.76	-0.464	<0.001	Abundance change	0.056/ 0.102
scale(Years passed):	-0.865	-1.593	-0.172	0.01	Abundance change	
scale(PSR)	-0.587	-1.423	0.343	0.198	Abundance change	
scale(Years):scale(PSR)	-0.445	-1.352	0.521	0.396	Abundance change	
(Intercept)	-4.549	-5.736	-3.464	<0.001	Total biomass change	0.104/ 0.192
scale(Years passed):	-2.84	-4.031	-1.395	<0.001	Total biomass change	
scale(PSR)	-0.731	-1.942	0.687	0.314	Total biomass change	
scale(Years):scale(PSR)	-0.537	-1.861	0.729	0.414	Total biomass change	
(Intercept)	-3.596	-3.675	-3.514	<0.001	Species lost	0.19/ 0.299
scale(Years passed):	-0.325	-0.431	-0.211	<0.001	Species lost	
scale(PSR)	-0.254	-0.329	-0.184	<0.001	Species lost	
scale(Years):scale(PSR)	-0.041	-0.147	0.061	0.396	Species lost	
(Intercept)	2.811	2.715	2.898	<0.001	Species gained	0.086/ 0.29
scale(Years passed):	-0.144	-0.281	0.048	0.1	Species gained	
scale(PSR)	0.197	0.1	0.293	<0.001	Species gained	
scale(Years):scale(PSR)	-0.001	-0.113	0.09	0.996	Species gained	
(Intercept)	-1.402	-1.698	-1.1	<0.001	Species richness	0.139/ 0.218
scale(Years passed):	-0.881	-1.203	-0.419	<0.001	Species richness	
scale(PSR)	-0.161	-0.393	0.129	0.272	Species richness	
scale(Years):scale(PSR)	-0.116	-0.39	0.153	0.382	Species richness	

Supplementary Table 6 Median estimates, 95% confidence intervals (2.5th–97.5th percentiles; CI low and CI high), two-sided p-values and median R^2 values across 1000 subsamples without replacement (see methods) of the restricted moving average analysis of overall arthropod responses (square root transformed) in the Jena Experiment. LUI = land use intesity, m/c = marginal/conditional.

Parameter	Estimate (median)	CI low	CI high	p-value	Response	m/c R^2
(Intercept)	-18.085	-18.448	-17.693	<0.001	Species richness loss	0.038/ 0.519
scale(Years passed):	-1.911	-2.759	-1.086	<0.001	Species richness loss	
scale(LUI)	1.188	0.792	1.521	<0.001	Species richness loss	
scale(Years):scale(LUI)	0.198	-0.556	0.923	0.59	Species richness loss	
(Intercept)	16.694	16.144	17.234	<0.001	Species richness gain	0.014/ 0.391
scale(Years passed):	0.245	-0.889	1.205	0.672	Species richness gain	
scale(LUI)	-0.886	-1.456	-0.302	0.004	Species richness gain	
scale(Years):scale(LUI)	-0.044	-0.613	0.478	0.844	Species richness gain	
(Intercept)	4.267	3.498	5.047	<0.001	Species identity loss	0.024/ 0.221
scale(Years passed):	-1.647	-2.532	-0.707	<0.001	Species identity loss	
scale(LUI)	-0.259	-1.038	0.554	0.526	Species identity loss	
scale(Years):scale(LUI)	-0.175	-1.12	0.705	0.68	Species identity loss	
(Intercept)	-3.501	-4.218	-2.722	<0.001	Species identity gain	0.05/ 0.225
scale(Years passed):	2.131	0.818	3.232	0.002	Species identity gain	
scale(LUI)	0.819	0.073	1.492	0.028	Species identity gain	
scale(Years):scale(LUI)	0.302	-0.557	1.092	0.494	Species identity gain	
(Intercept)	-1.552	-2.342	-0.716	<0.001	Abundance change	0.015/ 0.25
scale(Years passed):	-1.396	-2.691	-0.296	0.012	Abundance change	
scale(LUI)	-0.154	-1.011	0.695	0.69	Abundance change	
scale(Years):scale(LUI)	0.165	-0.837	1.074	0.76	Abundance change	
(Intercept)	-2.05	-3.285	-0.786	0.004	Total biomass change	0.011/ 0.452
scale(Years passed):	-2.205	-3.98	-0.545	0.01	Total biomass change	
scale(LUI)	0.645	-0.599	1.865	0.288	Total biomass change	
scale(Years):scale(LUI)	0.259	-1	1.537	0.718	Total biomass change	
(Intercept)	-3.251	-3.303	-3.202	<0.001	Species lost	0.112/ 0.529
scale(Years passed):	-0.329	-0.392	-0.262	<0.001	Species lost	
scale(LUI)	0.159	0.113	0.202	<0.001	Species lost	
scale(Years):scale(LUI)	0.014	-0.042	0.074	0.644	Species lost	
(Intercept)	3.131	3.07	3.188	<0.001	Species gained	0.043/ 0.325
scale(Years passed):	0.149	0.028	0.224	0.03	Species gained	
scale(LUI)	-0.121	-0.178	-0.062	<0.001	Species gained	
scale(Years):scale(LUI)	-0.003	-0.066	0.056	0.896	Species gained	
(Intercept)	-0.165	-0.327	0.004	0.054	Species richness	0.008/ 0.421
scale(Years passed):	-0.227	-0.404	-0.039	0.012	Species richness	
scale(LUI)	0.078	-0.089	0.234	0.36	Species richness	
scale(Years):scale(LUI)	0	-0.163	0.168	0.996	Species richness	

Supplementary Table 7 Median predictions and 95% confidence intervals (CI) for each available time span in the restricted moving average analysis in the Jena Experiment. Percentages were calculated based on the sum of the components per time span (year).

Year	Predicted median	CI low	CI high	% of total biomass change	CI low	CI high	Component
0	-88.871	-102.871	-76.462	732.836	630.51	848.281	Species richness loss
2	-103.463	-111.926	-95.403	283.026	260.978	306.177	Species richness loss
4	-118.525	-126.074	-111.631	192.333	181.146	204.583	Species richness loss
5	-126.554	-136.236	-117.004	169.032	156.276	181.963	Species richness loss
6	-134.766	-148.16	-121.282	152.275	137.039	167.409	Species richness loss
7	-143.339	-160.549	-125.35	140.066	122.487	156.883	Species richness loss
0	77.124	62.02	92.549	-635.969	-763.165	-511.421	Species richness gain
2	67.082	59.002	75.372	-183.505	-206.182	-161.402	Species richness gain
4	57.973	51.99	64.775	-94.074	-105.112	-84.365	Species richness gain
5	53.612	46.665	62.477	-71.607	-83.447	-62.328	Species richness gain
6	49.365	40.728	61.128	-55.778	-69.07	-46.019	Species richness gain
7	45.48	35.261	61.092	-44.441	-59.697	-34.456	Species richness gain
0	1.814	0.052	5.823	-14.958	-48.017	-0.429	Species identity loss
2	1.263	0.177	3.344	-3.455	-9.148	-0.484	Species identity loss
4	0.87	0.134	2.196	-1.412	-3.563	-0.217	Species identity loss
5	0.681	0.033	2.183	-0.91	-2.916	-0.044	Species identity loss
6	0.519	-0.007	2.46	-0.586	-2.78	0.008	Species identity loss
7	0.391	-0.139	2.848	-0.382	-2.783	0.136	Species identity loss
0	-2.195	-6.253	-0.214	18.1	1.765	51.563	Species identity gain
2	-1.068	-2.986	-0.122	2.922	0.334	8.168	Species identity gain
4	-0.376	-1.28	-0.016	0.61	0.026	2.077	Species identity gain
5	-0.161	-0.882	0.015	0.215	-0.02	1.178	Species identity gain
6	-0.035	-0.755	0.201	0.04	-0.227	0.853	Species identity gain
7	0.001	-0.626	0.678	-0.001	-0.663	0.612	Species identity gain
0	0.001	-1.448	1.752	-0.008	-14.447	11.94	Abundance change
2	-0.37	-2.01	0.072	1.012	-0.197	5.498	Abundance change
4	-1.567	-3.535	-0.373	2.543	0.605	5.736	Abundance change
5	-2.448	-4.948	-0.816	3.27	1.09	6.609	Abundance change
6	-3.585	-7.359	-1.266	4.051	1.43	8.315	Abundance change
7	-4.87	-10.697	-1.612	4.759	1.575	10.453	Abundance change
0	-12.234	-32.025	6.119	100.882	-50.458	264.08	Species richness turnover
2	-36.361	-47.855	-24.395	99.467	66.733	130.909	Species richness turnover
4	-60.265	-71.912	-49.183	97.793	79.81	116.693	Species richness turnover
5	-72.584	-87.062	-58.376	96.947	77.97	116.284	Species richness turnover
6	-85.28	-102.873	-66.981	96.359	75.683	116.238	Species richness turnover
7	-97.928	-119.442	-74.221	95.692	72.526	116.714	Species richness turnover
0	-0.313	-4.278	3.582	2.581	-29.537	35.277	Species identity turnover
2	0.158	-1.727	2.167	-0.432	-5.928	4.724	Species identity turnover
4	0.429	-0.49	1.825	-0.696	-2.961	0.795	Species identity turnover
5	0.48	-0.288	1.979	-0.641	-2.643	0.385	Species identity turnover
6	0.43	-0.199	2.29	-0.486	-2.588	0.225	Species identity turnover
7	0.455	-0.26	2.816	-0.445	-2.752	0.254	Species identity turnover
0	-12.925	-33.185	6.316	106.58	-52.082	273.646	Species turnover

Year	Predicted median	CI low	CI high	% of total biomass change	CI low	CI high	Component
2	-36.212	-47.876	-24	99.059	65.653	130.966	Species turnover
4	-59.737	-71.609	-48.743	96.936	79.096	116.201	Species turnover
5	-72.04	-86.425	-57.729	96.22	77.106	115.433	Species turnover
6	-84.612	-102.344	-65.723	95.605	74.262	115.64	Species turnover
7	-97.492	-119.107	-73.653	95.266	71.971	116.387	Species turnover

Supplementary Table 8 Median predictions and 95% confidence intervals (CI) for each available time span in the restricted moving average analysis in the Biodiversity Exploratories. Percentages were calculated based on the sum of the components per time span (year).

Year	Predicted median	CI low	CI high	% of total biomass change	CI low	CI high	Component
0	-253.524	-285.001	-222.986	-1252.4	-1101.55	-1407.9	Species richness loss
1	-274.966	-299.845	-252.192	-12402.6	-11375.4	-13524.8	Species richness loss
2	-296.835	-314.744	-281.923	1719.487	1633.105	1823.229	Species richness loss
3	-320.071	-332.049	-308.359	832.283	801.828	863.429	Species richness loss
4	-344.023	-358.469	-328.617	561.881	536.719	585.475	Species richness loss
5	-368.529	-392.795	-345.265	432.084	404.808	460.535	Species richness loss
6	-394.203	-430.04	-360.4	355.522	325.036	387.843	Species richness loss
7	-419.839	-469.96	-374.155	305.258	272.042	341.7	Species richness loss
8	-446.806	-510.825	-388.547	268.404	233.406	306.861	Species richness loss
9	-474.853	-553.918	-404.492	241.513	205.727	281.726	Species richness loss
0	270.43	230.539	312.5	1335.919	1543.744	1138.858	Species richness gain
1	272.727	242.315	303.356	12301.62	13683.18	10929.86	Species richness gain
2	274.995	253.815	297.582	-1592.97	-1723.81	-1470.28	Species richness gain
3	277.712	259.756	297.074	-722.136	-772.484	-675.445	Species richness gain
4	280.306	259.663	300.393	-457.814	-490.622	-424.099	Species richness gain
5	283.011	253.174	310.43	-331.818	-363.966	-296.836	Species richness gain
6	286.276	245.865	322.383	-258.185	-290.749	-221.74	Species richness gain
7	289.35	236.668	336.131	-210.381	-244.395	-172.077	Species richness gain
8	292.37	227.519	349.359	-175.631	-209.866	-136.674	Species richness gain
9	295.301	219.067	362.985	-150.192	-184.616	-111.419	Species richness gain
0	37.996	23.811	53.7	187.699	265.277	117.626	Species identity loss
1	31.175	20.446	42.849	1406.18	1932.747	922.237	Species identity loss
2	24.945	17.196	33.801	-144.5	-195.8	-99.612	Species identity loss
3	19.603	13.412	26.99	-50.974	-70.182	-34.875	Species identity loss
4	14.948	9.325	21.509	-24.414	-35.13	-15.23	Species identity loss
5	10.917	5.622	17.912	-12.8	-21.001	-6.592	Species identity loss
6	7.586	2.522	14.941	-6.842	-13.475	-2.275	Species identity loss
7	4.727	0.58	12.359	-3.437	-8.986	-0.422	Species identity loss
8	2.573	-0.005	10.672	-1.546	-6.411	0.003	Species identity loss
9	1.056	-0.917	8.902	-0.537	-4.528	0.466	Species identity loss
0	-34.659	-50.337	-20.154	-171.215	-99.56	-248.664	Species identity gain
1	-26.51	-36.882	-16.994	-1195.76	-766.531	-1663.6	Species identity gain
2	-19.511	-26.577	-13.29	113.022	76.985	153.954	Species identity gain
3	-13.707	-19.092	-8.92	35.642	23.195	49.645	Species identity gain
4	-8.84	-14.731	-4.595	14.438	7.505	24.06	Species identity gain
5	-4.892	-11.873	-1.366	5.736	1.602	13.921	Species identity gain
6	-2.219	-9.85	-0.032	2.001	0.029	8.883	Species identity gain
7	-0.583	-7.86	0.722	0.424	-0.525	5.715	Species identity gain
8	0	-6.317	3.689	0	-2.216	3.795	Species identity gain
9	0.55	-4.922	9.331	-0.28	-4.746	2.503	Species identity gain
0	0	-2.671	3.217	0	15.892	-13.195	Abundance change
1	-0.209	-3.174	0.908	-9.427	40.956	-143.166	Abundance change
2	-0.857	-3.891	0.024	4.964	-0.139	22.54	Abundance change

Year	Predicted median	CI low	CI high	% of total biomass change	CI low	CI high	Component
3	-1.994	-5.046	-0.309	5.185	0.803	13.121	Abundance change
4	-3.618	-7.235	-1.171	5.909	1.913	11.817	Abundance change
5	-5.798	-11.427	-2.087	6.798	2.447	13.398	Abundance change
6	-8.32	-17.894	-2.86	7.504	2.579	16.138	Abundance change
7	-11.191	-26.247	-3.445	8.137	2.505	19.084	Abundance change
8	-14.605	-36.291	-4.1	8.773	2.463	21.801	Abundance change
9	-18.67	-47.454	-4.521	9.496	2.299	24.135	Abundance change
0	15.489	-29.113	67.829	76.515	335.074	-143.818	Species richness turnover
1	-3.028	-37.771	34.166	-136.581	1541.092	-1703.7	Species richness turnover
2	-21.686	-48.237	4.939	125.621	-28.61	279.424	Species richness turnover
3	-42.314	-64.491	-19.359	110.029	50.339	167.696	Species richness turnover
4	-63.957	-93.729	-37.144	104.459	60.666	153.084	Species richness turnover
5	-86.06	-129.472	-46.845	100.902	54.924	151.8	Species richness turnover
6	-109.25	-169.23	-56.942	98.53	51.355	152.624	Species richness turnover
7	-132.307	-210.846	-66.523	96.198	48.368	153.302	Species richness turnover
8	-156.899	-253.58	-73.67	94.252	44.255	152.33	Species richness turnover
9	-181.709	-297.815	-81.505	92.418	41.454	151.47	Species richness turnover
0	3.116	-13.456	20.948	15.393	103.483	-66.472	Species identity turnover
1	4.347	-7.406	16.636	196.076	750.383	-334.055	Species identity turnover
2	5.463	-2.891	14.164	-31.646	-82.048	16.747	Species identity turnover
3	5.957	-0.517	13.179	-15.49	-34.269	1.344	Species identity turnover
4	6.03	-0.402	13.168	-9.849	-21.507	0.657	Species identity turnover
5	5.755	-1.193	12.685	-6.747	-14.873	1.399	Species identity turnover
6	4.829	-1.779	11.862	-4.355	-10.698	1.604	Species identity turnover
7	3.619	-2.157	11.022	-2.631	-8.014	1.568	Species identity turnover
8	2.612	-2.34	9.911	-1.569	-5.954	1.406	Species identity turnover
9	2.662	-2.964	11.843	-1.354	-6.023	1.508	Species identity turnover
0	19.663	-32.434	74.429	97.135	367.678	-160.223	Species turnover
1	1.93	-37.602	42.216	87.055	1904.195	-1696.08	Species turnover
2	-15.98	-45.196	10.867	92.568	-62.95	261.808	Species turnover
3	-36.624	-62.095	-11.038	95.234	28.702	161.466	Species turnover
4	-57.937	-89.352	-27.604	94.627	45.085	145.936	Species turnover
5	-80.969	-124.852	-40.229	94.933	47.167	146.384	Species turnover
6	-103.873	-164.372	-51.284	93.681	46.252	148.243	Species turnover
7	-128.54	-206.763	-59.344	93.459	43.148	150.334	Species turnover
8	-153.125	-251.184	-68.22	91.985	40.981	150.89	Species turnover
9	-178.289	-294.831	-76.423	90.679	38.869	149.953	Species turnover

Supplementary Table 9 Median estimates, 95% confidence intervals (CI) and two-sided p-values of the restricted moving average analysis of overall arthropod biomass and species richness loss (square root transformed) in the Jena Experiment when including the change in plant biomass in the predictors. PSR = plant species richness.

Parameter	Estimate (median)	CI low	CI high	p-value	Response
(Intercept)	-4.549	-5.742	-3.464	<0.001	Total biomass change
scale(Years passed)	-2.743	-3.919	-1.315	<0.001	Total biomass change
scale(Plant biomass change)	0.826	-0.263	2.039	0.168	Total biomass change
scale(PSR)	-0.681	-1.882	0.702	0.342	Total biomass change
scale(Years):scale(PSR)	-0.485	-1.833	0.777	0.47	Total biomass change
(Intercept)	-1.403	-1.699	-1.102	<0.001	Species turnover
scale(Years passed)	-0.88	-1.199	-0.415	<0.001	Species turnover
scale(Plant biomass change)	0.01	-0.267	0.281	0.942	Species turnover
scale(PSR)	-0.16	-0.393	0.13	0.276	Species turnover
scale(Years):scale(PSR)	-0.115	-0.385	0.151	0.388	Species turnover

Supplementary Table 10 Analysis results based on mean species biomass (excluding abundance): Median estimates, 95% confidence intervals (CI) and two-sided p-values of the restricted moving average analysis of overall arthropod responses (square root transformed) in the Jena Experiment. PSR = plant species richness.

Parameter	Estimate (median)	CI low	CI high	p-value	Response
(Intercept)	-8.317	-8.61	-8.055	<0.001	Species richness loss
scale(Years passed):	-0.715	-1.068	-0.32	<0.001	Species richness loss
scale(PSR)	-0.689	-0.961	-0.45	<0.001	Species richness loss
scale(Years):scale(PSR)	-0.092	-0.451	0.238	0.59	Species richness loss
(Intercept)	6.194	5.885	6.501	<0.001	Species richness gain
scale(Years passed):	-0.345	-0.875	0.359	0.266	Species richness gain
scale(PSR)	0.551	0.285	0.861	<0.001	Species richness gain
scale(Years):scale(PSR)	0.004	-0.328	0.298	0.974	Species richness gain
(Intercept)	-1.61	-2.013	-1.221	<0.001	Species identity loss
scale(Years passed):	0.211	-0.232	0.687	0.386	Species identity loss
scale(PSR)	-0.249	-0.72	0.238	0.302	Species identity loss
scale(Years):scale(PSR)	0.032	-0.466	0.519	0.902	Species identity loss
(Intercept)	1.188	0.766	1.553	<0.001	Species identity gain
scale(Years passed):	-0.447	-0.856	0.047	0.08	Species identity gain
scale(PSR)	0.262	-0.203	0.719	0.278	Species identity gain
scale(Years):scale(PSR)	-0.036	-0.501	0.43	0.894	Species identity gain
(Intercept)	-3.209	-4.144	-2.324	<0.001	Total biomass change
scale(Years passed):	-1.749	-2.883	-0.506	0.006	Total biomass change
scale(PSR)	-0.305	-1.112	0.635	0.534	Total biomass change
scale(Years):scale(PSR)	-0.251	-1.185	0.642	0.61	Total biomass change

Supplementary Table 11 Analysis results based on mean species biomass (excluding abundance): Median estimates, 95% confidence intervals (CI) and two-sided p-values of the restricted moving average analysis of overall arthropod responses (square root transformed) in the Biodiversity Exploratories. LUI = land use intensity.

**Parameter**	**Estimate (median)**	**CI low**	**CI high**	**p-value**	**Response**
(Intercept)	-11.212	-11.46	-10.956	<0.001	Species richness loss
scale(Years passed):	-1.135	-1.554	-0.753	<0.001	Species richness loss
scale(LUI)	0.979	0.72	1.211	<0.001	Species richness loss
scale(Years):scale(LUI)	0.101	-0.235	0.449	0.542	Species richness loss
(Intercept)	10.924	10.625	11.268	<0.001	Species richness gain
scale(Years passed):	0.742	0.217	1.142	0.02	Species richness gain
scale(LUI)	-0.7	-0.994	-0.39	<0.001	Species richness gain
scale(Years):scale(LUI)	0.001	-0.309	0.329	0.994	Species richness gain
(Intercept)	-1.753	-2.214	-1.308	<0.001	Species identity loss
scale(Years passed):	0.269	-0.209	0.772	0.282	Species identity loss
scale(LUI)	-0.07	-0.498	0.36	0.75	Species identity loss
scale(Years):scale(LUI)	0.048	-0.459	0.499	0.826	Species identity loss
(Intercept)	1.716	1.271	2.13	<0.001	Species identity gain
scale(Years passed):	-0.23	-0.739	0.269	0.366	Species identity gain
scale(LUI)	0.229	-0.189	0.65	0.272	Species identity gain
scale(Years):scale(LUI)	0.016	-0.464	0.532	0.942	Species identity gain
(Intercept)	-0.307	-1.207	0.55	0.454	Total biomass change
scale(Years passed):	-0.429	-1.56	0.637	0.466	Total biomass change
scale(LUI)	0.545	-0.302	1.363	0.192	Total biomass change
scale(Years):scale(LUI)	0.167	-0.661	1.084	0.69	Total biomass change

Supplementary Table 12 Median estimates, 95% confidence intervals (CI) and two-sided p-values of the within-year between-replicates change over time of overall arthropod responses (square root transformed) in the Jena Experiment. PSR = plant species richness.

Parameter	Estimate (median)	CI low	CI high	p-value	Response
(Intercept)	-7.494	-7.65	-7.345	<0.001	Species richness loss
scale(Year)	1.516	1.38	1.658	<0.001	Species richness loss
scale(PSR)	-0.87	-1.032	-0.72	<0.001	Species richness loss
scale(Year):scale(PSR)	0.177	0.005	0.339	0.04	Species richness loss
(Intercept)	7.498	7.342	7.646	<0.001	Species richness gain
scale(Year)	-1.519	-1.656	-1.378	<0.001	Species richness gain
scale(PSR)	0.872	0.711	1.023	<0.001	Species richness gain
scale(Year):scale(PSR)	-0.164	-0.336	-0.002	0.046	Species richness gain
(Intercept)	1.278	1.059	1.494	<0.001	Species identity loss
scale(Year)	-0.495	-0.73	-0.285	<0.001	Species identity loss
scale(PSR)	0.723	0.458	0.938	<0.001	Species identity loss
scale(Year):scale(PSR)	-0.218	-0.472	0.014	0.08	Species identity loss
(Intercept)	-1.283	-1.502	-1.065	<0.001	Species identity gain
scale(Year)	0.504	0.269	0.715	<0.001	Species identity gain
scale(PSR)	-0.711	-0.977	-0.497	<0.001	Species identity gain
scale(Year):scale(PSR)	0.232	-0.023	0.463	0.094	Species identity gain

Supplementary Table 13 Median estimates, 95% confidence intervals (CI) and two-sided p-values of the within-year between-replicates change over time of overall arthropod responses (square root transformed) in the Biodiversity Exploratories. LUI = land use intensity.

Parameter	Estimate (median)	CI low	CI high	p-value	Response
(Intercept)	-13.938	-14.102	-13.773	<0.001	Species richness loss
scale(Year)	1.635	1.464	1.8	<0.001	Species richness loss
scale(LUI)	0.879	0.699	1.048	<0.001	Species richness loss
scale(Year):scale(LUI)	-0.239	-0.401	-0.075	0.004	Species richness loss
(Intercept)	13.943	13.779	14.11	<0.001	Species richness gain
scale(Year)	-1.639	-1.81	-1.474	<0.001	Species richness gain
scale(LUI)	-0.884	-1.063	-0.714	<0.001	Species richness gain
scale(Year):scale(LUI)	0.227	0.065	0.391	0.01	Species richness gain
(Intercept)	5.694	5.457	5.936	<0.001	Species identity loss
scale(Year)	-1.939	-2.188	-1.696	<0.001	Species identity loss
scale(LUI)	-0.308	-0.546	-0.068	0.02	Species identity loss
scale(Year):scale(LUI)	0.261	0.053	0.482	0.016	Species identity loss
(Intercept)	-5.697	-5.934	-5.456	<0.001	Species identity gain
scale(Year)	1.942	1.693	2.185	<0.001	Species identity gain
scale(LUI)	0.304	0.066	0.546	0.01	Species identity gain
scale(Year):scale(LUI)	-0.259	-0.467	-0.038	0.028	Species identity gain

Supplementary Table 14 Analysis results based on mean species biomass (excluding abundance): Median estimates, 95% confidence intervals (CI) and two-sided p-values of the within-year between-replicates change over time of overall arthropod responses (square root transformed) in the Jena Experiment. PSR = plant species richness.

Parameter	Estimate (median)	CI low	CI high	p-value	Response
(Intercept)	-7.494	-7.65	-7.345	<0.001	Species richness loss
scale(Year)	1.516	1.38	1.658	<0.001	Species richness loss
scale(PSR)	-0.87	-1.032	-0.72	<0.001	Species richness loss
scale(Year):scale(PSR)	0.177	0.005	0.339	0.04	Species richness loss
(Intercept)	7.498	7.342	7.646	<0.001	Species richness gain
scale(Year)	-1.519	-1.656	-1.378	<0.001	Species richness gain
scale(PSR)	0.872	0.711	1.023	<0.001	Species richness gain
scale(Year):scale(PSR)	-0.164	-0.336	-0.002	0.046	Species richness gain
(Intercept)	1.278	1.059	1.494	<0.001	Species identity loss
scale(Year)	-0.495	-0.73	-0.285	<0.001	Species identity loss
scale(PSR)	0.723	0.458	0.938	<0.001	Species identity loss
scale(Year):scale(PSR)	-0.218	-0.472	0.014	0.08	Species identity loss
(Intercept)	-1.283	-1.502	-1.065	<0.001	Species identity gain
scale(Year)	0.504	0.269	0.715	<0.001	Species identity gain
scale(PSR)	-0.711	-0.977	-0.497	<0.001	Species identity gain
scale(Year):scale(PSR)	0.232	-0.023	0.463	0.094	Species identity gain

Supplementary Table 15 Analysis results based on mean species biomass (excluding abundance): Median estimates, 95% confidence intervals (CI) and two-sided p-values of the within-year between-replicates change over time of overall arthropod responses (square root transformed) in the Biodiversity Exploratories. LUI = land use intensity.

Parameter	Estimate (median)	CI low	CI high	p-value	Response
(Intercept)	-9.352	-9.49	-9.208	<0.001	Species richness loss
scale(Year)	0.261	0.128	0.404	<0.001	Species richness loss
scale(LUI)	0.696	0.57	0.835	<0.001	Species richness loss
scale(Year):scale(LUI)	-0.19	-0.313	-0.064	0.004	Species richness loss
(Intercept)	9.355	9.217	9.5	<0.001	Species richness gain
scale(Year)	-0.265	-0.397	-0.123	<0.001	Species richness gain
scale(LUI)	-0.706	-0.831	-0.563	<0.001	Species richness gain
scale(Year):scale(LUI)	0.179	0.055	0.305	0.01	Species richness gain
(Intercept)	-1.519	-1.74	-1.32	<0.001	Species identity loss
scale(Year)	0.479	0.261	0.719	<0.001	Species identity loss
scale(LUI)	0.187	-0.008	0.397	0.062	Species identity loss
scale(Year):scale(LUI)	0.282	0.085	0.478	0.006	Species identity loss
(Intercept)	1.523	1.299	1.72	<0.001	Species identity gain
scale(Year)	-0.484	-0.702	-0.246	<0.001	Species identity gain
scale(LUI)	-0.197	-0.387	0.014	0.066	Species identity gain
scale(Year):scale(LUI)	-0.289	-0.489	-0.092	0.002	Species identity gain

Supplementary Table 16 Model summary for the relationship of sampling coverage with sampling year and plant species richness (PSR) in the Jena Experiment. DF = degrees of freedom.

Parameter	Estimate	Std. error	DF	p-value
(Intercept)	0.869	0.002	77.848	<0.001
scale(Year)	-0.011	0.001	477.564	<0.001
scale(PSR)	0.008	0.002	77.783	<0.001
scale(Year):scale(PSR)	0	0.001	477.181	0.937

Supplementary Table 17 Model summary for the relationship of sampling coverage with sampling year and land-use intensity (LUI) in the Biodiversity Exploratories. DF = degrees of freedom.

Parameter	Estimate	Std. error	DF	p-value
(Intercept)	0.895	0.002	150.783	<0.001
scale(Year)	-0.006	0.001	1360.215	<0.001
scale(LUI)	0	0.001	315.16	0.972
scale(Year):scale(LUI)	-0.001	0.001	1411.538	0.14

Supplementary Table 18 Model summary for the relationship of species abundance with aerial mobility in the Jena Experiment (see Supplementary Fig. 5). DF = degrees of freedom.

Parameter	Estimate	Std. error	Z value	p-value
(Intercept)	0.261	0.045	5.799	<0.001
Aerial mobility B	-0.202	0.047	-4.269	<0.001
Aerial mobility C	0.917	0.032	28.386	<0.001
Aerial mobility D	0.331	0.033	9.941	<0.001

Supplementary Table 19 Model summary for the relationship of species abundance with Dispersal ability in the Biodiversity Exploratories (see Supplementary Fig. 5). DF = degrees of freedom.

Parameter	Estimate	Std. error	Z value	p-value
(Intercept)	0.75	0.023	32.037	<0.001
Dispersal ability 0.25	0.224	0.018	12.276	<0.001
Dispersal ability 0.5	0.474	0.016	30.17	<0.001
Dispersal ability 0.75	-0.21	0.016	-13.071	<0.001
Dispersal ability 1	0.24	0.014	16.675	<0.001

Supplementary Table 20 Median predictions and 95% confidence intervals (CI) for each available time span in the restricted moving average analysis in the Jena Experiment, when including only species that occur in at least 30% of all plots per year. Percentages were calculated based on the sum of the components per time span (year).

**Year**	**Predicted median**	**CI low**	**CI high**	**% of total biomass change**	**CI low**	**CI high**	**Component**
0	-36.57	-42.804	-30.636	354.611	297.068	415.058	Species richness loss
2	-47.159	-51.315	-43.152	199.177	182.253	216.726	Species richness loss
4	-59.19	-63.603	-54.89	152.173	141.119	163.519	Species richness loss
5	-65.643	-72.152	-59.694	138.692	126.123	152.444	Species richness loss
6	-72.54	-81.279	-64.479	128.533	114.25	144.018	Species richness loss
7	-79.783	-91.216	-69.249	120.755	104.811	138.058	Species richness loss
0	26.547	17.03	33.617	-257.418	-325.976	-165.135	Species richness gain
2	23.183	18.928	27.069	-97.913	-114.325	-79.94	Species richness gain
4	20.434	17.832	23.304	-52.535	-59.913	-45.845	Species richness gain
5	19.008	15.556	24.036	-40.16	-50.783	-32.866	Species richness gain
6	17.628	13.178	24.909	-31.234	-44.137	-23.35	Species richness gain
7	16.298	10.981	26.296	-24.668	-39.801	-16.62	Species richness gain
0	3.727	1.249	7.463	-36.139	-72.367	-12.108	Species identity loss
2	2.929	1.41	4.967	-12.37	-20.977	-5.954	Species identity loss
4	2.18	0.941	3.844	-5.605	-9.882	-2.42	Species identity loss
5	1.88	0.63	3.757	-3.972	-7.938	-1.331	Species identity loss
6	1.589	0.296	3.874	-2.816	-6.864	-0.524	Species identity loss
7	1.349	0.063	4.102	-2.042	-6.208	-0.095	Species identity loss
0	-4.017	-7.273	-1.408	38.947	13.657	70.528	Species identity gain
2	-2.337	-4.04	-0.962	9.872	4.061	17.064	Species identity gain
4	-1.109	-1.98	-0.473	2.85	1.217	5.09	Species identity gain
5	-0.655	-1.389	-0.179	1.384	0.379	2.935	Species identity gain
6	-0.322	-0.993	-0.015	0.57	0.027	1.759	Species identity gain
7	-0.104	-0.805	0.048	0.157	-0.073	1.218	Species identity gain
0	0	-1.496	1.551	0	-15.043	14.508	Abundance change
2	-0.292	-1.869	0.095	1.234	-0.401	7.893	Abundance change
4	-1.212	-2.866	-0.264	3.116	0.679	7.369	Abundance change
5	-1.92	-3.948	-0.606	4.056	1.28	8.341	Abundance change
6	-2.792	-5.607	-0.881	4.948	1.561	9.935	Abundance change
7	-3.831	-7.83	-1.003	5.798	1.518	11.851	Abundance change
0	-9.992	-20.676	-1.534	96.89	14.878	200.489	Species richness turnover
2	-23.928	-29.559	-18.375	101.06	77.608	124.843	Species richness turnover
4	-38.79	-44.424	-32.429	99.728	83.374	114.212	Species richness turnover
5	-46.749	-54.407	-38.028	98.772	80.347	114.952	Species richness turnover
6	-54.89	-64.909	-42.828	97.259	75.887	115.012	Species richness turnover
7	-63.278	-76.149	-47.969	95.773	72.602	115.255	Species richness turnover
0	-0.425	-4.156	3.696	4.12	-35.839	40.304	Species identity turnover
2	0.512	-1.739	2.937	-2.163	-12.403	7.346	Species identity turnover
4	1.096	-0.2	2.748	-2.818	-7.066	0.514	Species identity turnover
5	1.176	-0.073	2.957	-2.484	-6.247	0.154	Species identity turnover
6	1.221	-0.044	3.366	-2.163	-5.965	0.078	Species identity turnover
7	1.155	-0.035	3.827	-1.749	-5.792	0.054	Species identity turnover
0	-10.257	-21.674	-0.836	99.463	8.109	210.171	Species turnover

Year	Predicted median	CI low	CI high	% of total biomass change	CI low	CI high	Component
2	-23.4	-29.378	-17.415	98.831	73.551	124.076	Species turnover
4	-37.729	-43.707	-31.482	96.999	80.939	112.367	Species turnover
5	-45.458	-53.268	-36.857	96.044	77.871	112.546	Species turnover
6	-53.42	-63.902	-41.627	94.654	73.759	113.228	Species turnover
7	-61.95	-74.996	-46.416	93.763	70.253	113.509	Species turnover

Supplementary Table 21 Median predictions and 95% confidence intervals (CI) for each available time span in the restricted moving average analysis in the Biodiversity Exploratories, when including only species that occur in at least 30% of all plots per year. Percentages were calculated based on the sum of the components per time span (year).

Year	Predicted median	CI low	CI high	% of total biomass change	CI low	CI high	Component
0	-46.671	-58.171	-36.89	-328.189	-259.405	-409.055	Species richness loss
1	-58.7	-68.335	-50.25	-1063.6	-910.49	-1238.19	Species richness loss
2	-72.309	-79.768	-65.076	1272.027	1144.783	1403.228	Species richness loss
3	-87.287	-93.867	-80.522	451.599	416.597	485.641	Species richness loss
4	-103.604	-111.975	-95.314	293.151	269.693	316.837	Species richness loss
5	-121.32	-135.153	-110.354	227.275	206.732	253.189	Species richness loss
6	-140.34	-159.531	-124.962	192.971	171.826	219.358	Species richness loss
7	-161.045	-186.465	-139.98	172.849	150.239	200.132	Species richness loss
8	-182.998	-215.725	-155.796	160.649	136.769	189.378	Species richness loss
9	-206.301	-247.416	-172.364	152.279	127.229	182.627	Species richness loss
0	61.71	45.514	84.158	433.938	591.793	320.049	Species richness gain
1	63.718	51.377	79.928	1154.523	1448.25	930.911	Species richness gain
2	65.779	56.657	76.278	-1157.14	-1341.84	-996.684	Species richness gain
3	67.637	60.717	74.951	-349.935	-387.777	-314.132	Species richness gain
4	69.415	60.686	77.9	-196.413	-220.419	-171.714	Species richness gain
5	71.426	57.932	83.995	-133.806	-157.353	-108.526	Species richness gain
6	73.66	53.699	91.605	-101.284	-125.959	-73.837	Species richness gain
7	75.623	50.088	98.852	-81.166	-106.097	-53.759	Species richness gain
8	77.646	46.039	107.605	-68.163	-94.463	-40.417	Species richness gain
9	79.778	42.694	117.238	-58.888	-86.538	-31.514	Species richness gain
0	10.486	5.321	16.577	73.737	116.567	37.418	Species identity loss
1	8.083	4.325	12.363	146.457	224.005	78.375	Species identity loss
2	5.915	3.049	9.135	-104.048	-160.696	-53.637	Species identity loss
3	4.104	1.954	6.633	-21.232	-34.317	-10.11	Species identity loss
4	2.66	0.928	4.887	-7.526	-13.828	-2.625	Species identity loss
5	1.504	0.224	3.697	-2.818	-6.926	-0.421	Species identity loss
6	0.683	-0.009	2.757	-0.939	-3.791	0.013	Species identity loss
7	0.178	-0.416	2.07	-0.191	-2.222	0.447	Species identity loss
8	0	-1.565	1.532	0	-1.345	1.374	Species identity loss
9	-0.155	-3.391	1.042	0.115	-0.769	2.503	Species identity loss
0	-11.297	-16.634	-6.763	-79.441	-47.559	-116.97	Species identity gain
1	-7.33	-10.836	-4.243	-132.807	-76.881	-196.336	Species identity gain
2	-4.202	-6.537	-2.209	73.915	38.868	114.992	Species identity gain
3	-1.922	-3.526	-0.765	9.943	3.956	18.243	Species identity gain
4	-0.517	-1.649	-0.036	1.464	0.102	4.665	Species identity gain
5	-0.003	-0.544	0.301	0.006	-0.565	1.019	Species identity gain
6	0.362	-0.056	1.752	-0.498	-2.41	0.077	Species identity gain
7	1.585	0.061	4.746	-1.702	-5.094	-0.065	Species identity gain
8	3.745	0.493	8.975	-3.287	-7.879	-0.433	Species identity gain
9	6.723	1.342	14.716	-4.962	-10.862	-0.99	Species identity gain
0	-0.006	-2.285	1.655	-0.045	11.636	-16.07	Abundance change
1	-0.253	-2.747	0.348	-4.575	6.308	-49.782	Abundance change

Year	Predicted median	CI low	CI high	% of total biomass change	CI low	CI high	Component
2	-0.867	-3.437	-0.004	15.25	0.075	60.465	Abundance change
3	-1.86	-4.222	-0.403	9.625	2.086	21.843	Abundance change
4	-3.295	-5.902	-1.285	9.324	3.636	16.7	Abundance change
5	-4.987	-8.928	-2.218	9.343	4.156	16.725	Abundance change
6	-7.091	-12.992	-3.067	9.75	4.217	17.864	Abundance change
7	-9.513	-18.435	-3.901	10.21	4.187	19.786	Abundance change
8	-12.304	-24.971	-4.673	10.802	4.102	21.921	Abundance change
9	-15.52	-32.121	-5.006	11.456	3.695	23.71	Abundance change
0	15.51	-2.847	38.919	109.066	273.678	-20.017	Species richness turnover
1	5.126	-9.528	22.197	92.887	402.191	-172.647	Species richness turnover
2	-6.5	-18.39	6.258	114.34	-110.09	323.502	Species richness turnover
3	-19.602	-29.74	-8.858	101.416	45.828	153.865	Species richness turnover
4	-34.258	-47.734	-21.93	96.935	62.051	135.064	Species richness turnover
5	-50.358	-68.914	-32.785	94.338	61.418	129.101	Species richness turnover
6	-67.385	-93.25	-43.386	92.656	59.657	128.221	Species richness turnover
7	-85.539	-119.727	-53.832	91.809	57.777	128.502	Species richness turnover
8	-105.551	-148.765	-66.049	92.66	57.982	130.596	Species richness turnover
9	-126.444	-180.162	-79.162	93.333	58.432	132.984	Species richness turnover
0	-0.755	-7.217	5.577	-5.306	39.216	-50.746	Species identity turnover
1	0.763	-3.62	5.146	13.82	93.246	-65.585	Species identity turnover
2	1.734	-1.359	4.941	-30.504	-86.915	23.91	Species identity turnover
3	2.172	-0.02	4.731	-11.237	-24.476	0.104	Species identity turnover
4	2.039	0.408	4.362	-5.77	-12.344	-1.155	Species identity turnover
5	1.468	0.257	3.69	-2.751	-6.912	-0.482	Species identity turnover
6	1.203	0.149	3.743	-1.654	-5.146	-0.205	Species identity turnover
7	2	0.231	5.523	-2.147	-5.928	-0.248	Species identity turnover
8	3.829	0.457	9.102	-3.361	-7.99	-0.401	Species identity turnover
9	6.305	0.906	13.854	-4.654	-10.226	-0.669	Species identity turnover
0	14.786	-3.389	38.233	103.974	268.852	-23.828	Species turnover
1	5.868	-9.018	22.999	106.326	416.727	-163.395	Species turnover
2	-4.727	-16.545	7.72	83.159	-135.807	291.043	Species turnover
3	-17.415	-28.082	-6.647	90.098	34.389	145.289	Species turnover
4	-32.202	-45.331	-19.875	91.118	56.236	128.265	Species turnover
5	-48.724	-67.048	-30.891	91.277	57.869	125.604	Species turnover
6	-66.028	-92.024	-41.759	90.789	57.42	126.535	Species turnover
7	-83.785	-118.514	-51.069	89.926	54.812	127.2	Species turnover
8	-102.057	-145.763	-60.551	89.593	53.156	127.961	Species turnover
9	-120.453	-175.661	-70.447	88.911	51.999	129.662	Species turnover

Supplementary Table 22 Median predictions and 95% confidence intervals (CI) for each available time span for low diversity plots (monoculture) and high diversity plots (60 plant species) in the restricted moving average analysis in the Jena Experiment. Percentages were calculated based on the sum of the components per time span (year). PSR = plant species richness.

Year	PSR	Predicted median	CI low	CI high	% of total biomass change	CI low	CI high	Component
0	1	-77.21	-92.929	-63.787	675.208	557.822	812.672	Species richness loss
0	60	-187.814	-295.234	-107.277	805.999	460.377	1266.99	Species richness loss
2	1	-89.163	-98.041	-80.576	279.569	252.645	307.406	Species richness loss
2	60	-222.858	-288.118	-165.975	333.96	248.719	431.754	Species richness loss
4	1	-101.755	-109.738	-94.234	192.001	177.81	207.065	Species richness loss
4	60	-259.425	-320.926	-211.1	207.746	169.048	256.996	Species richness loss
5	1	-108.579	-119.385	-98.22	169.203	153.06	186.042	Species richness loss
5	60	-278.869	-361.143	-216.293	175.096	135.806	226.755	Species richness loss
6	1	-115.384	-130.107	-100.939	153.722	134.478	173.337	Species richness loss
6	60	-296.747	-413.278	-215.199	152.034	110.254	211.737	Species richness loss
7	1	-122.457	-141.846	-103.114	141.915	119.498	164.385	Species richness loss
7	60	-316.634	-470.042	-207.117	134.717	88.121	199.986	Species richness loss
0	1	65.941	50.854	82.034	-576.659	-717.394	-444.722	Species richness gain
0	60	168.051	105.053	279.268	-721.187	-1198.47	-450.833	Species richness gain
2	1	57.463	49.044	66.054	-180.174	-207.111	-153.777	Species richness gain
2	60	149.847	108.059	217.378	-224.55	-325.748	-161.93	Species richness gain
4	1	49.471	43.715	55.799	-93.347	-105.287	-82.486	Species richness gain
4	60	131.006	96.542	180.408	-104.909	-144.47	-77.31	Species richness gain
5	1	45.558	38.78	54.324	-70.995	-84.655	-60.432	Species richness gain
5	60	122.597	84.084	173.548	-76.976	-108.967	-52.795	Species richness gain
6	1	41.959	33.574	53.718	-55.901	-71.567	-44.73	Species richness gain
6	60	113.861	71.88	172.828	-58.335	-88.546	-36.827	Species richness gain
7	1	38.448	28.482	53.163	-44.557	-61.61	-33.008	Species richness gain
7	60	105.746	55.545	176.561	-44.991	-75.121	-23.632	Species richness gain
0	1	0.596	-0.266	3.969	-5.212	-34.709	2.326	Species identity loss
0	60	26.455	-0.494	93.153	-113.531	-399.764	2.12	Species identity loss
2	1	0.301	-0.061	1.622	-0.944	-5.086	0.191	Species identity loss
2	60	25.988	1.891	63.701	-38.944	-95.458	-2.834	Species identity loss
4	1	0.095	-0.075	0.826	-0.179	-1.559	0.142	Species identity loss
4	60	25.163	4.886	60.623	-20.15	-48.547	-3.913	Species identity loss
5	1	0.036	-0.259	0.872	-0.056	-1.359	0.404	Species identity loss
5	60	24.795	2.78	69.369	-15.568	-43.555	-1.746	Species identity loss
6	1	0.009	-0.672	1.157	-0.012	-1.541	0.895	Species identity loss
6	60	24.348	0.698	83.671	-12.474	-42.868	-0.358	Species identity loss
7	1	0	-1.453	1.427	0	-1.654	1.684	Species identity loss
7	60	24.62	-0.071	102.235	-10.475	-43.497	0.03	Species identity loss
0	1	-0.766	-4.299	0.105	6.699	-0.918	37.595	Species identity gain
0	60	-29.997	-96.814	-0.017	128.731	0.073	415.475	Species identity gain
2	1	-0.349	-1.949	0.04	1.094	-0.125	6.111	Species identity gain
2	60	-15.807	-46.719	-0.46	23.687	0.689	70.01	Species identity gain
4	1	-0.102	-0.835	0.044	0.192	-0.083	1.576	Species identity gain
4	60	-6.291	-23.729	0.002	5.038	-0.002	19.002	Species identity gain

Year	PSR	Predicted median	CI low	CI high	% of total biomass change	CI low	CI high	Component
5	1	-0.039	-0.67	0.157	0.061	-0.245	1.044	Species identity gain
5	60	-3.22	-20.086	0.941	2.022	-0.591	12.612	Species identity gain
6	1	-0.003	-0.658	0.439	0.004	-0.585	0.877	Species identity gain
6	60	-1.091	-17.481	5.889	0.559	-3.017	8.956	Species identity gain
7	1	0.008	-0.775	0.928	-0.009	-1.075	0.898	Species identity gain
7	60	-0.11	-20.061	15.978	0.047	-6.798	8.535	Species identity gain
0	1	0.004	-2.177	2.079	-0.035	-18.181	19.038	Abundance change
0	60	0.003	-48.27	63.458	-0.013	-272.329	207.15	Abundance change
2	1	-0.145	-2.042	0.316	0.455	-0.991	6.403	Abundance change
2	60	-3.902	-41.434	11	5.847	-16.484	62.09	Abundance change
4	1	-0.706	-2.445	-0.016	1.332	0.03	4.613	Abundance change
4	60	-15.329	-54.269	-0.031	12.275	0.025	43.458	Abundance change
5	1	-1.147	-3.397	-0.11	1.787	0.171	5.294	Abundance change
5	60	-24.569	-72.411	-0.823	15.426	0.517	45.465	Abundance change
6	1	-1.641	-5.005	-0.182	2.186	0.242	6.668	Abundance change
6	60	-35.556	-102.656	-1.268	18.217	0.65	52.594	Abundance change
7	1	-2.288	-7.291	-0.217	2.652	0.251	8.45	Abundance change
7	60	-48.659	-145.174	-1.62	20.703	0.689	61.766	Abundance change

Supplementary Table 23 Median predictions and 95% confidence intervals (CI) for each available time span for low land use plots (LUI = 0.5) and high land use plots (LUI = 3.5) in the restricted moving average analysis in the Jena Experiment. Percentages were calculated based on the sum of the components per time span (year). LUI = land-use intensity.

Year	LUI	Predicted median	CI low	CI high	% of total biomass change	CI low	CI high	Component
0	0.5	-315.088	-389.9	-242.753	-11408	-8789.03	-14116.6	Species richness loss
0	3.5	-167.717	-262.537	-94.107	-453.399	-254.405	-709.732	Species richness loss
1	0.5	-343.756	-402.951	-285.881	1670.665	1389.391	1958.354	Species richness loss
1	3.5	-179.307	-253.811	-119.774	-677.167	-452.336	-958.537	Species richness loss
2	0.5	-373.899	-418.24	-330.071	830.591	733.23	929.092	Species richness loss
2	3.5	-190.001	-243.847	-147.446	-1305.58	-1013.17	-1675.58	Species richness loss
3	0.5	-404.561	-437.46	-370.607	562.562	515.347	608.31	Species richness loss
3	3.5	-202.819	-242.045	-172.233	16651.81	14140.64	19872.33	Species richness loss
4	0.5	-436.268	-470.926	-402.397	434.422	400.694	468.933	Species richness loss
4	3.5	-215.651	-255.366	-182.201	1137.58	961.128	1347.08	Species richness loss
5	0.5	-470.016	-517.348	-422.555	355.217	319.348	390.988	Species richness loss
5	3.5	-228.675	-289.268	-177.749	632.066	491.304	799.547	Species richness loss
6	0.5	-504.441	-572.811	-436.944	307.234	266.124	348.875	Species richness loss
6	3.5	-242.265	-329.221	-171.178	459	324.317	623.749	Species richness loss
7	0.5	-539.755	-636.965	-449.789	273.612	228.007	322.89	Species richness loss
7	3.5	-256.982	-376.484	-160.915	396.412	248.222	580.752	Species richness loss
8	0.5	-575.905	-705.825	-461.934	247.094	198.195	302.837	Species richness loss
8	3.5	-272.612	-427.815	-152.292	365.422	204.139	573.463	Species richness loss
9	0.5	-614.748	-779.036	-474.473	226.183	174.572	286.629	Species richness loss
9	3.5	-288.492	-483.497	-143.86	345.293	172.184	578.692	Species richness loss
0	0.5	324.336	253.297	401.924	11742.8	14551.92	9170.782	Species richness gain
0	3.5	188.996	118.183	294.627	510.924	796.483	319.491	Species richness gain
1	0.5	328.43	269.814	392.041	-1596.18	-1905.33	-1311.3	Species richness gain
1	3.5	190.908	129.149	272.157	720.979	1027.822	487.741	Species richness gain
2	0.5	333.34	282.726	384.206	-740.492	-853.488	-628.057	Species richness gain
2	3.5	192.034	136.874	256.965	1319.549	1765.718	940.521	Species richness gain
3	0.5	336.503	291.844	379.511	-467.924	-527.729	-405.824	Species richness gain
3	3.5	193.496	142.727	250.86	-15886.4	-20596.1	-11718.1	Species richness gain
4	0.5	340.323	297.209	383.032	-338.883	-381.411	-295.951	Species richness gain
4	3.5	193.573	146.286	255.448	-1021.12	-1347.51	-771.673	Species richness gain
5	0.5	343.597	292.052	395.925	-259.675	-299.222	-220.72	Species richness gain
5	3.5	193.83	140.716	265.364	-535.753	-733.475	-388.944	Species richness gain
6	0.5	348.621	283.909	410.733	-212.33	-250.16	-172.917	Species richness gain
6	3.5	192.955	133.817	278.653	-365.577	-527.942	-253.533	Species richness gain
7	0.5	353.566	274.018	431.124	-179.229	-218.545	-138.905	Species richness gain
7	3.5	194.044	120.411	292.687	-299.326	-451.489	-185.742	Species richness gain
8	0.5	358.358	260.33	450.281	-153.755	-193.195	-111.696	Species richness gain
8	3.5	195.752	106.899	312.854	-262.395	-419.364	-143.292	Species richness gain
9	0.5	363.2	248.503	473.853	-133.632	-174.344	-91.431	Species richness gain
9	3.5	196.525	94.35	335.226	-235.218	-401.228	-112.926	Species richness gain
0	0.5	39.012	10.937	78.434	1412.455	2839.754	395.981	Species identity loss
0	3.5	35.717	3.983	104.997	96.556	283.845	10.767	Species identity loss

Year	LUI	Predicted median	CI low	CI high	% of total biomass change	CI low	CI high	Component
1	0.5	33.559	11.659	62.836	-163.098	-305.385	-56.663	Species identity loss
1	3.5	27.659	3.342	77.502	104.456	292.692	12.621	Species identity loss
2	0.5	28.626	12.043	51.644	-63.591	-114.724	-26.753	Species identity loss
2	3.5	20.205	2.778	55.564	138.837	381.804	19.089	Species identity loss
3	0.5	24.195	9.979	44.086	-33.644	-61.304	-13.876	Species identity loss
3	3.5	13.546	1.385	41.186	-1112.15	-3381.45	-113.711	Species identity loss
4	0.5	19.658	6.984	39.021	-19.575	-38.856	-6.954	Species identity loss
4	3.5	8.51	0.138	31.977	-44.891	-168.682	-0.728	Species identity loss
5	0.5	15.898	3.324	37.641	-12.015	-28.447	-2.512	Species identity loss
5	3.5	4.824	-0.665	26.389	-13.334	-72.94	1.838	Species identity loss
6	0.5	12.395	0.865	36.697	-7.549	-22.351	-0.527	Species identity loss
6	3.5	1.972	-5.394	25.726	-3.736	-48.741	10.22	Species identity loss
7	0.5	9.922	-0.011	39.281	-5.03	-19.912	0.006	Species identity loss
7	3.5	0.394	-15.256	25.884	-0.608	-39.928	23.533	Species identity loss
8	0.5	7.307	-1.514	40.665	-3.135	-17.447	0.65	Species identity loss
8	3.5	-0.029	-30.737	26.506	0.039	-35.53	41.201	Species identity loss
9	0.5	5.199	-4.908	43.556	-1.913	-16.025	1.806	Species identity loss
9	3.5	-0.863	-49.233	27.841	1.033	-33.323	58.926	Species identity loss
0	0.5	-45.968	-89.16	-16.799	-1664.3	-608.219	-3228.1	Species identity gain
0	3.5	-19.239	-69.616	-0.003	-52.01	-0.008	-188.197	Species identity gain
1	0.5	-38.826	-69.251	-16.624	188.696	80.793	336.562	Species identity gain
1	3.5	-11.385	-43.067	0.01	-42.996	0.038	-162.646	Species identity gain
2	0.5	-32.859	-54.442	-15.979	72.994	35.496	120.939	Species identity gain
2	3.5	-5.212	-24.883	0.095	-35.814	0.653	-170.982	Species identity gain
3	0.5	-26.937	-44.254	-12.793	37.457	17.789	61.537	Species identity gain
3	3.5	-1.535	-13.228	0.944	126.026	-77.504	1086.043	Species identity gain
4	0.5	-21.457	-39.352	-9.031	21.366	8.993	39.185	Species identity gain
4	3.5	-0.028	-7.207	4.166	0.148	-21.976	38.018	Species identity gain
5	0.5	-16.805	-36.793	-4.358	12.7	3.294	27.806	Species identity gain
5	3.5	0.776	-4.037	11.847	-2.145	-32.746	11.158	Species identity gain
6	0.5	-12.653	-36.383	-0.966	7.706	0.588	22.159	Species identity gain
6	3.5	3.634	-2.631	25.212	-6.885	-47.767	4.985	Species identity gain
7	0.5	-9.322	-37.759	0.02	4.726	-0.01	19.141	Species identity gain
7	3.5	8.79	-2.125	44.627	-13.559	-68.84	3.278	Species identity gain
8	0.5	-6.42	-40.167	1.6	2.755	-0.686	17.234	Species identity gain
8	3.5	15.833	-0.94	72.562	-21.223	-97.265	1.26	Species identity gain
9	0.5	-3.961	-41.743	5.912	1.457	-2.175	15.358	Species identity gain
9	3.5	24.994	-0.658	107.189	-29.915	-128.293	0.788	Species identity gain
0	0.5	0.47	-7.15	15.19	17.017	549.964	-258.87	Abundance change
0	3.5	-0.766	-38.687	14.567	-2.071	39.38	-104.585	Abundance change
1	0.5	0.017	-7.136	7.56	-0.083	-36.742	34.681	Abundance change
1	3.5	-1.396	-30.827	6.705	-5.272	25.322	-116.421	Abundance change
2	0.5	-0.224	-7.352	3.133	0.498	-6.96	16.332	Abundance change
2	3.5	-2.473	-26.695	2.466	-16.993	16.945	-183.433	Abundance change
3	0.5	-1.114	-9.265	0.599	1.549	-0.833	12.883	Abundance change
3	3.5	-3.906	-23.3	0.62	320.69	-50.903	1912.972	Abundance change

Year	LUI	Predicted median	CI low	CI high	% of total biomass change	CI low	CI high	Component
4	0.5	-2.681	-12.757	0.02	2.67	-0.02	12.703	Abundance change
4	3.5	-5.361	-25.348	0.089	28.28	-0.469	133.713	Abundance change
5	0.5	-4.992	-19.151	-0.061	3.773	0.046	14.473	Abundance change
5	3.5	-6.934	-34.11	0.152	19.166	-0.42	94.281	Abundance change
6	0.5	-8.11	-28.573	-0.157	4.939	0.096	17.403	Abundance change
6	3.5	-9.077	-45.618	0.655	17.197	-1.241	86.429	Abundance change
7	0.5	-11.681	-41.28	-0.215	5.921	0.109	20.926	Abundance change
7	3.5	-11.073	-62.036	1.925	17.081	-2.969	95.695	Abundance change
8	0.5	-16.411	-58.264	-0.306	7.041	0.131	24.998	Abundance change
8	3.5	-13.546	-83.999	3.464	18.158	-4.643	112.596	Abundance change
9	0.5	-21.482	-79.207	-0.284	7.904	0.104	29.143	Abundance change
9	3.5	-15.714	-113.531	5.774	18.808	-6.911	135.884	Abundance change

Supplementary Table 24 Median estimates, 95% confidence intervals (CI) and two-sided p-values of the restricted moving average analysis of arthropod responses per trophic guild (square root transformed) in the Jena Experiment. PSR = plant species richness.

Parameter	Trophic guild	Estimate (median)	CI low	CI high	p-value	Response
(Intercept)	herbivores	-6.889	-7.182	-6.601	<0.001	Species richness loss
scale(Years passed)	herbivores	-0.81	-1.186	-0.419	<0.001	Species richness loss
scale(PSR)	herbivores	-0.752	-1.061	-0.468	<0.001	Species richness loss
scale(Years):scale(PSR)	herbivores	-0.077	-0.53	0.32	0.716	Species richness loss
(Intercept)	herbivores	5.188	4.896	5.503	<0.001	Species richness gain
scale(Years passed)	herbivores	-0.418	-0.776	-0.005	0.048	Species richness gain
scale(PSR)	herbivores	0.768	0.427	1.089	<0.001	Species richness gain
scale(Years):scale(PSR)	herbivores	0.067	-0.274	0.456	0.716	Species richness gain
(Intercept)	herbivores	0.472	0.088	0.893	0.014	Species identity loss
scale(Years passed)	herbivores	-0.222	-0.716	0.248	0.374	Species identity loss
scale(PSR)	herbivores	0.404	-0.08	0.873	0.1	Species identity loss
scale(Years):scale(PSR)	herbivores	0.01	-0.596	0.662	0.974	Species identity loss
(Intercept)	herbivores	-0.363	-0.753	0.025	0.07	Species identity gain
scale(Years passed)	herbivores	0.399	-0.047	0.863	0.086	Species identity gain
scale(PSR)	herbivores	-0.249	-0.741	0.265	0.32	Species identity gain
scale(Years):scale(PSR)	herbivores	0.128	-0.461	0.649	0.646	Species identity gain
(Intercept)	herbivores	-0.64	-1.094	-0.222	0.002	Abundance change
scale(Years passed)	herbivores	-0.592	-1.101	-0.104	0.016	Abundance change
scale(PSR)	herbivores	-0.17	-0.771	0.416	0.606	Abundance change
scale(Years):scale(PSR)	herbivores	-0.139	-0.792	0.442	0.618	Abundance change
(Intercept)	herbivores	-2.612	-3.459	-1.711	<0.001	Total biomass change
scale(Years passed)	herbivores	-1.857	-2.714	-0.921	<0.001	Total biomass change
scale(PSR)	herbivores	-0.115	-1.039	0.873	0.792	Total biomass change
scale(Years):scale(PSR)	herbivores	-0.173	-1.116	0.815	0.772	Total biomass change
(Intercept)	predators	-7.419	-7.745	-7.089	<0.001	Species richness loss
scale(Years passed)	predators	-0.536	-1.036	-0.015	0.04	Species richness loss
scale(PSR)	predators	-1.006	-1.376	-0.614	<0.001	Species richness loss
scale(Years):scale(PSR)	predators	-0.128	-0.708	0.424	0.632	Species richness loss
(Intercept)	predators	5.018	4.667	5.429	<0.001	Species richness gain
scale(Years passed)	predators	-0.754	-1.218	-0.077	0.036	Species richness gain
scale(PSR)	predators	0.597	0.232	1.012	0.002	Species richness gain
scale(Years):scale(PSR)	predators	-0.164	-0.606	0.247	0.454	Species richness gain
(Intercept)	predators	0.486	0.031	0.964	0.04	Species identity loss
scale(Years passed)	predators	-0.225	-0.757	0.373	0.446	Species identity loss
scale(PSR)	predators	0.777	0.195	1.364	0.008	Species identity loss
scale(Years):scale(PSR)	predators	-0.016	-0.73	0.682	0.966	Species identity loss
(Intercept)	predators	-0.395	-0.82	0.016	0.062	Species identity gain
scale(Years passed)	predators	0.328	-0.152	0.804	0.18	Species identity gain
scale(PSR)	predators	-0.454	-0.931	0.057	0.092	Species identity gain
scale(Years):scale(PSR)	predators	0.295	-0.291	0.8	0.306	Species identity gain
(Intercept)	predators	-0.589	-1.09	-0.047	0.038	Abundance change
scale(Years passed)	predators	-0.424	-1.045	0.152	0.154	Abundance change
scale(PSR)	predators	-0.481	-1.187	0.307	0.268	Abundance change
scale(Years):scale(PSR)	predators	-0.389	-1.257	0.516	0.38	Abundance change

Parameter	Trophic guild	Estimate (median)	CI low	CI high	p-value	Response
(Intercept)	predators	-3.114	-3.97	-2.121	<0.001	Total biomass change
scale(Years passed)	predators	-1.752	-2.796	-0.524	0.012	Total biomass change
scale(PSR)	predators	-0.651	-1.611	0.47	0.266	Total biomass change
scale(Years):scale(PSR)	predators	-0.557	-1.606	0.521	0.322	Total biomass change

Supplementary Table 25 Air temperature (°C) and relative humidity (%) at 2 m height during the sampling days (24 h) in the Jena Experiment. Weather data were averaged across the sampling days in May and July to reflect the pooled analysis of samples from May and July (see methods). Mean and standard deviation (SD) of weather data across years are provided in the bottom rows.

Year	Air temperature	Relative humidity
2010	18.157	71.59
2012	20.616	66.221
2014	18.072	78.376
2016	19.199	68.206
2017	20.813	62.304
2019	16.585	69.806
2020	15.785	64.64
**Mean**	18.461	68.735
**SD**	1.758	4.884
**Year**	Air temperature	Relative humidity

Supplementary Table 26 Air temperature (°C) and relative humidity (%) at 2 m height during the sampling days (24 h) in the Biodiversity Exploratories. Weather data were averaged within each region and across the sampling days in June and August to reflect the pooled analysis of samples from June and August (see methods). Mean and standard deviation (SD) of weather data across years are provided in the bottom rows.

	Schwäbische Alb	Hainich	Schorfheide	Schwäbische Alb	Hainich	Schorfheide
2008	NA	NA	NA	NA	NA	NA
2009	14.955	15.255	17.056	81.053	88.541	77.416
2010	17.726	15.678	15.367	80.074	82.612	86.448
2011	17.729	15.897	16.454	86.925	85.079	84.692
2012	15.411	16.241	14.17	76.967	78.659	76.921
2013	NA	17.001	8.186	NA	77.103	85.652
2014	12.772	17.456	12.875	76.359	77.802	82.808
2015	21.916	NA	NA	68.858	NA	NA
2016	17.617	14.192	NA	79.204	86.976	NA
2017	19.063	20.235	NA	73.137	75.784	NA
2018	18.052	18.585	17.17	78.96	77.699	79.776
**Mean**	17.249	16.727	14.468	77.948	81.139	81.959
**SD**	2.345	1.643	2.758	4.546	4.25	3.405

Supplementary Figures

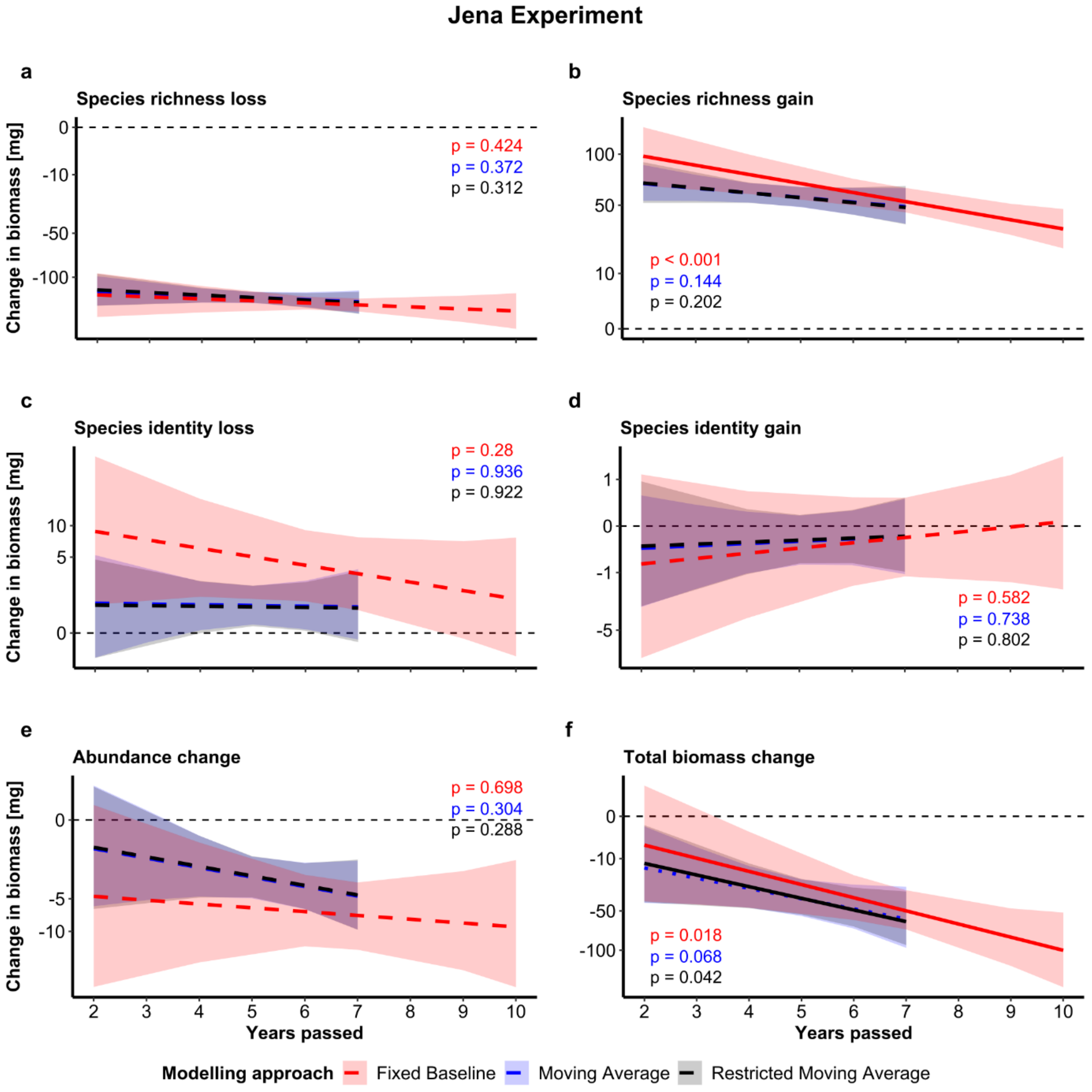

Supplementary Figure 1 ***Comparison of predicted regression lines according to the different modelling approaches for the Jena Experiment, when removing the control (community turnover between replicates within-years):*** *All panels are based on replicate-level (n = 160) median point estimates and predictions from 1,000 linear mixed-effects models drawing from shuffled data subsets, avoiding the reuse of sampling events in multiple pairwise comparisons. Fixed baseline comparison (2010 as the baseline; red), moving average comparison (2010-2019 as baseline; blue) and restricted moving average comparison (2010-2014 as baseline; black). Temporal biomass change was associated with a) species richness loss (assuming average biomass of lost species relative to their respective communities), b) species richness gain (assuming average biomass of gained species relative to their respective communities), c) species identity loss (deviation of observed biomass change associated with lost species from the expected biomass change associated with species richness loss), d) species identity gain (deviation of observed biomass change associated with gained species from the expected biomass change associated with species richness gain), and e) abundance change of persisting species. f) Total biomass change without partitioning. Solid lines indicate two-sided p < 0.05 (significant), dotted lines indicate p < 0.1 (marginally significant), dashed lines indicate non-significant relationships; exact p-values are provided in the panels.*

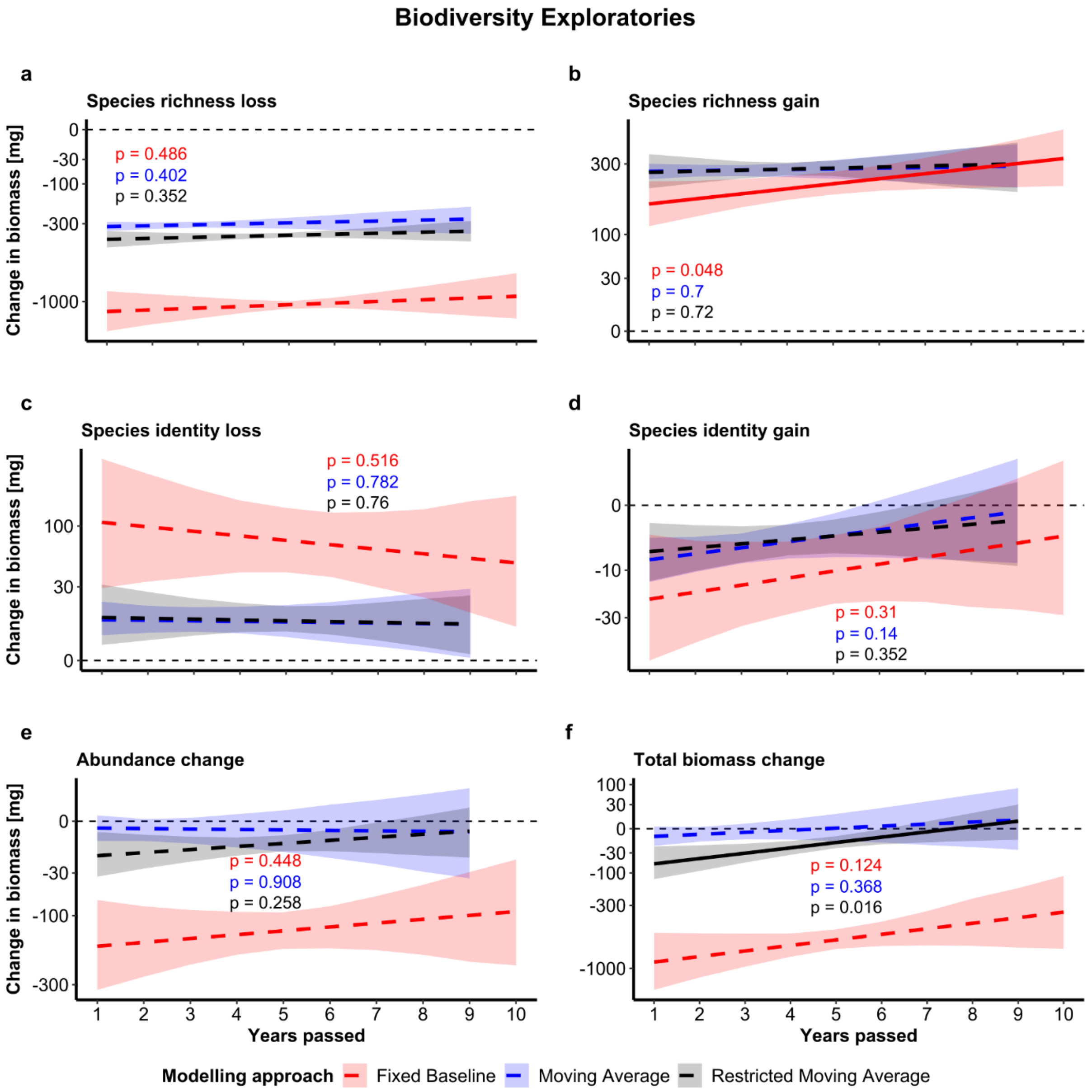

Supplementary Figure 2 ***Comparison of predicted regression lines according to the different modelling approaches for the Biodiversity Exploratories, when removing the control (community turnover between replicates within-years):*** *All panels are based on replicate-level (n = 300) median point estimates and predictions from 1,000 linear mixed-effects models drawing from shuffled data subsets, avoiding the reuse of sampling events in multiple pairwise comparisons. Fixed baseline comparison (2008 as the baseline; red), moving average comparison (2009-2017 as baseline; blue) and restricted moving average comparison (2008-2012 as baseline; black). Temporal biomass change was associated with a) species richness loss (assuming average biomass of lost species relative to their respective communities), b) species richness gain (assuming average biomass of gained species relative to their respective communities), c) species identity loss (deviation of observed biomass change associated with lost species from the expected biomass change associated with species richness loss), d) species identity gain (deviation of observed biomass change associated with gained species from the expected biomass change associated with species richness gain), and e) abundance change of persisting species. f) Total biomass change without partitioning. Solid lines indicate two-sided $p < 0.05$ (significant), dotted lines indicate $p < 0.1$ (marginally significant), dashed lines indicate non-significant relationships; exact p-values are provided in the panels.*

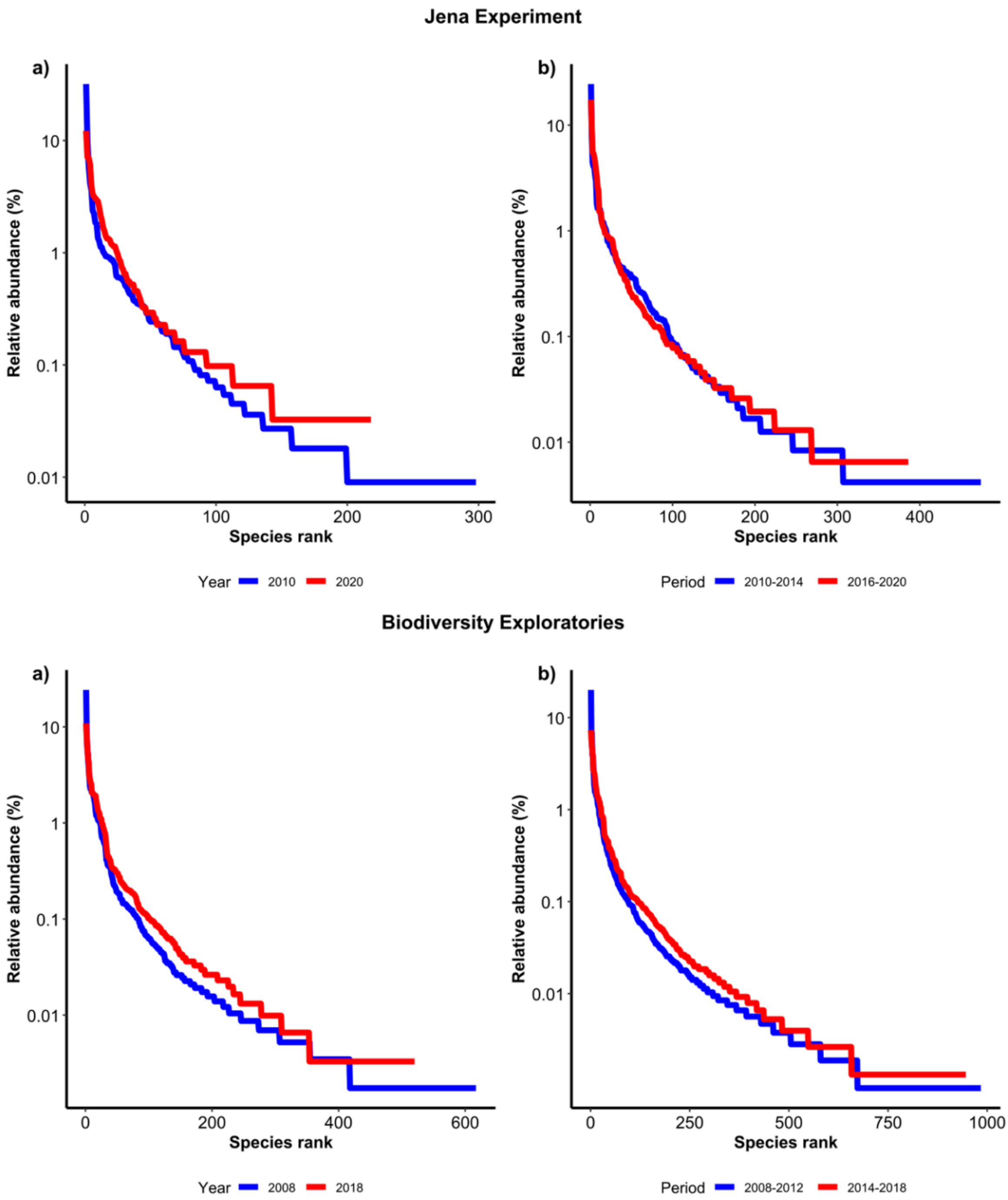

Supplementary Figure 3 ***Rank-abundance curves of relative arthropod abundances per replicate (n = 2) per plot (Jena Experiment: n = 80, Biodiversity Exploratories: n = 150) over time.*** *Curves of later years (red) generally show a higher relative abundance of common species and a reduced tail of rare species compared to earlier years (blue).*

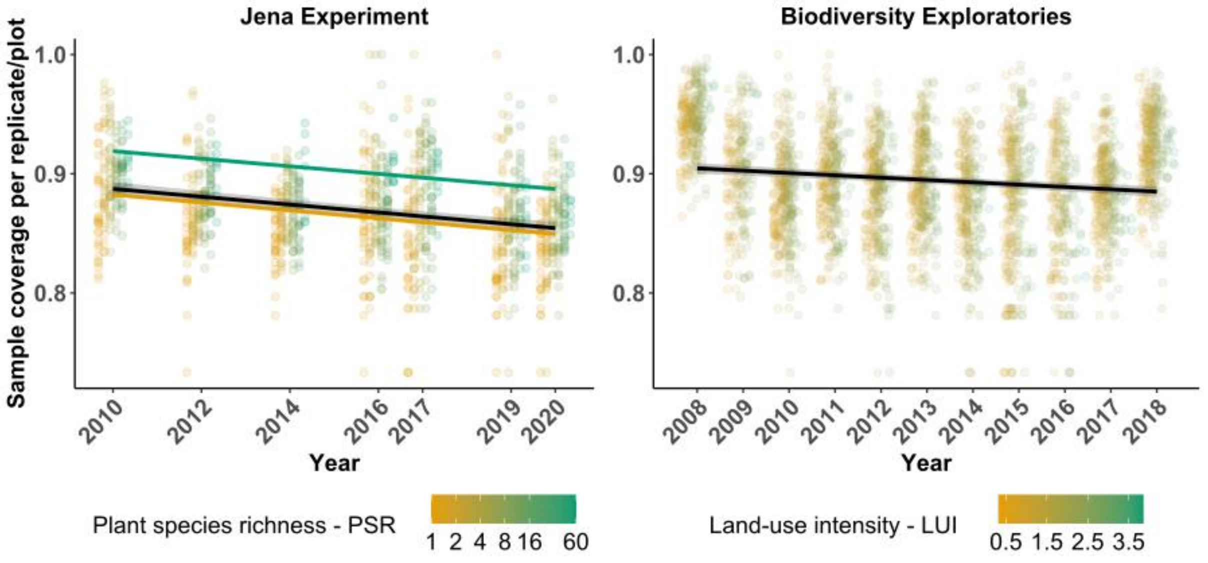

Supplementary Figure 4 ***Sample coverage per replicate per plot across time and plant species richness (PSR; Jena Experiment; n = 160) and land-use intensity (Biodiversity Exploratories; n = 300).*** *Derived from linear-mixed effects models. The datapoints are colored along the PSR and LUI gradients (see legend). Only significant relationships are shown (two-sided p-values in both cases < 0.001).*

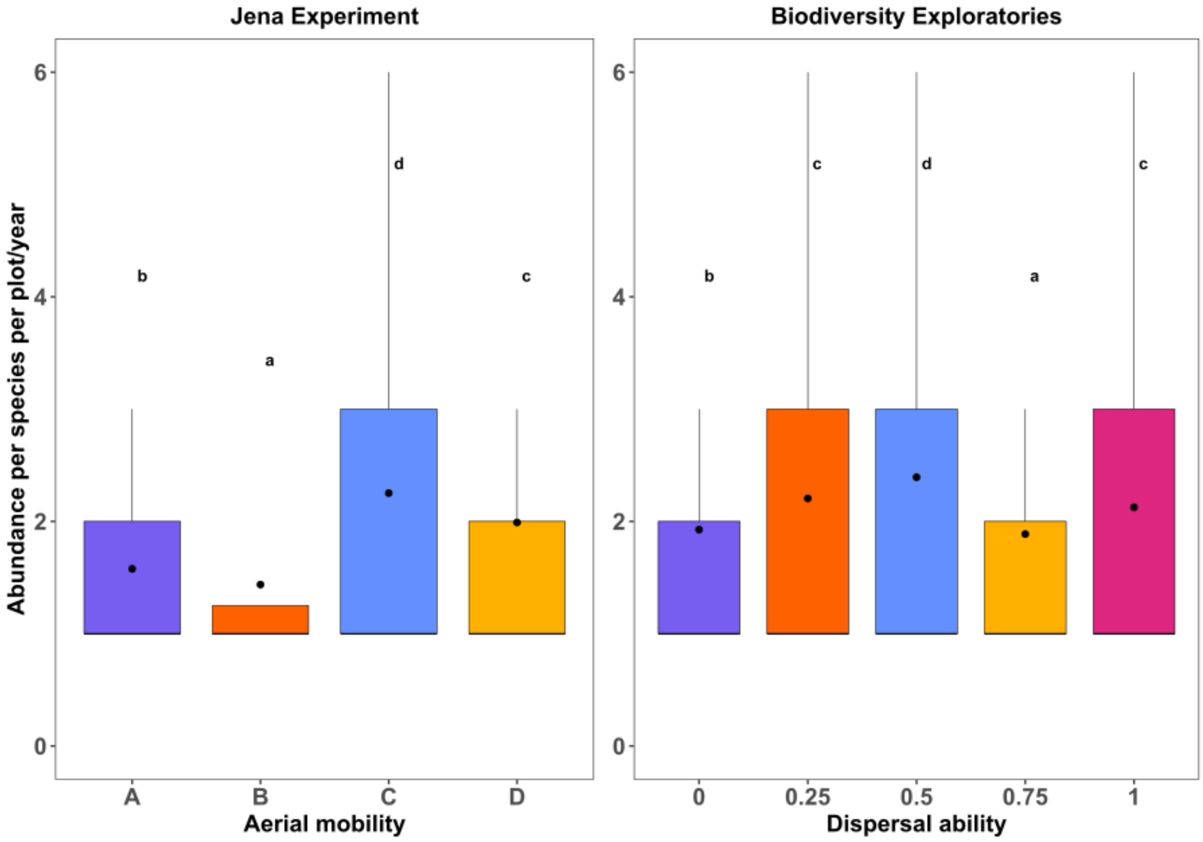

Supplementary Figure 5 ***Abundance per species per plot and year grouped according to their mobility*** *(measured as aerial mobility in the Jena Experiment*[7] *and dispersal ability in the Biodiversity Exploratories*[8]*, both in increasing order, i.e. A and 0 represent the lowest mobility respectively. Higher values are foremost associated with the presence and functionality of wings, or in the case of spiders with e.g. ballooning behavior). Significant differences between groups are indicated with letters. Linear mixed-effects models yielded two-sided p-values for both mobility categories below 0.001, pairwise comparisons were conducted with a Tukey test and Holm's correction for multiple comparisons.*

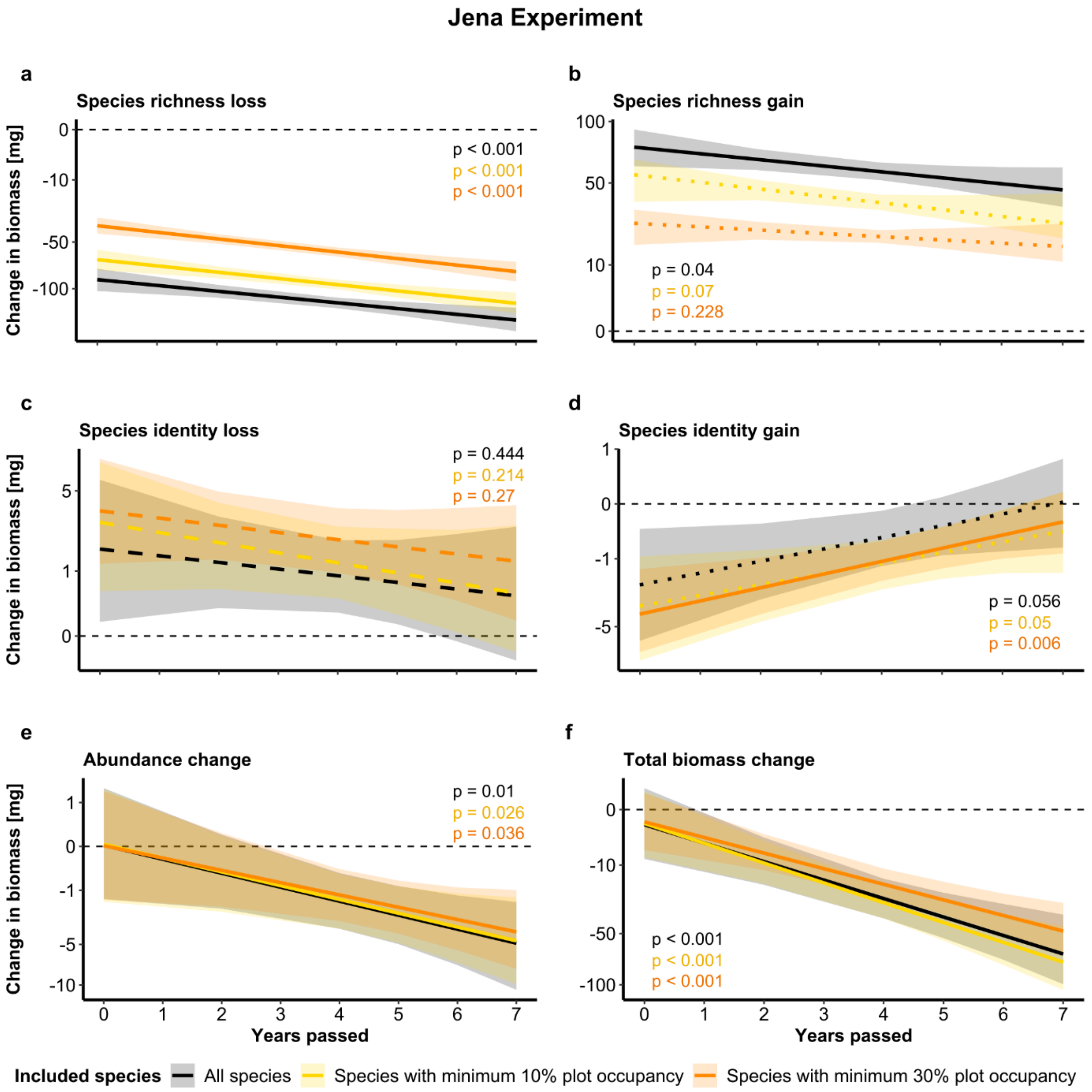

Supplementary Figure 6 ***Comparison of modelled temporal trends of partitioned biomass change (restricted moving average) in the Jena Experiment when including all species (black), only species that occur in at least 10% of all plots per year (yellow) and only species that occur in at least 30% of all plots per year (yellow).*** *All panels are based on replicate-level (n = 160) median point estimates and predictions from 1,000 linear mixed-effects models drawing from shuffled data subsets, avoiding the reuse of sampling events in multiple pairwise comparisons. Temporal biomass change was associated with a) species richness loss (assuming average biomass of lost species relative to their respective communities), b) species richness gain (assuming average biomass of gained species relative to their respective communities), c) species identity loss (deviation of observed biomass change associated with lost species from the expected biomass change associated with species richness loss), d) species identity gain (deviation of observed biomass change associated with gained species from the expected biomass change associated with species richness gain), and e) abundance change of persisting species. f) Total biomass change without partitioning. Solid lines indicate two-sided p < 0.05 (significant), dotted lines indicate p < 0.1 (marginally significant), dashed lines indicate non-significant relationships; exact p-values are provided in the panels.*

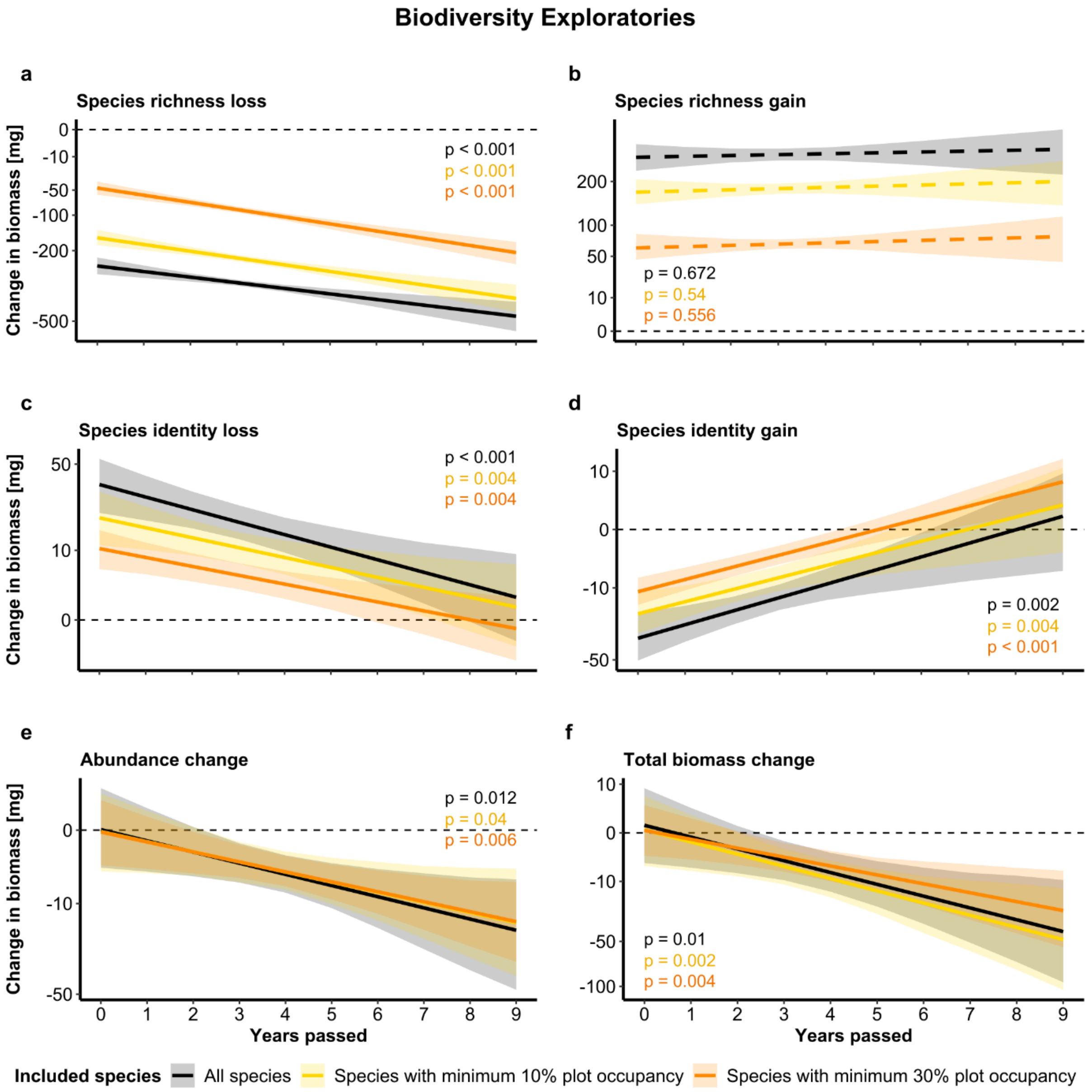

Supplementary Figure 7 ***Comparison of modelled temporal trends of partitioned biomass change (restricted moving average) in the Biodiversity Exploratories when including all species (black), only species that occur in at least 10% of all plots per year (yellow) and only species that occur in at least 30% of all plots per year (yellow).*** *All panels are based on replicate-level (n = 300) median point estimates and predictions from 1,000 linear mixed-effects models drawing from shuffled data subsets, avoiding the reuse of sampling events in multiple pairwise comparisons. Temporal biomass change was associated with a) species richness loss (assuming average biomass of lost species relative to their respective communities), b) species richness gain (assuming average biomass of gained species relative to their respective communities), c) species identity loss (deviation of observed biomass change associated with lost species from the expected biomass change associated with species richness loss), d) species identity gain (deviation of observed biomass change associated with gained species from the expected biomass change associated with species richness gain), and e) abundance change of persisting species. f) Total biomass change without partitioning. Solid lines indicate two-sided p < 0.05 (significant), dotted lines indicate p < 0.1 (marginally significant), dashed lines indicate non-significant relationships; exact p-values are provided in the panels.*

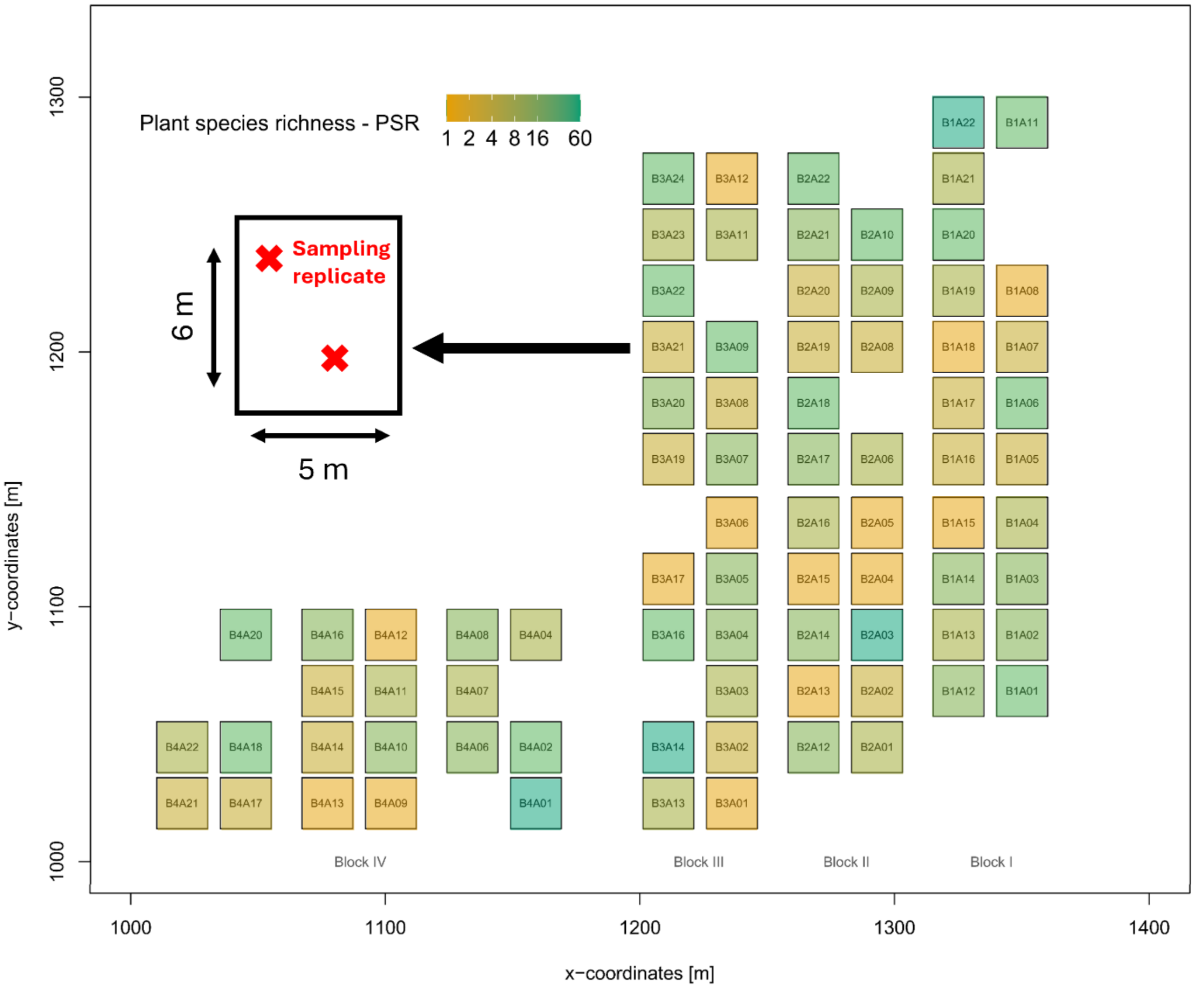

Supplementary Figure 8 ***Map of the Jena Experiment.*** *Plots are colored according to their sown plant species richness (PSR, see legend). Each plot has an area of 5x6 m and was sampled using suction sampling at two locations at each sampling (see inset graph and methods in the main manuscript).*

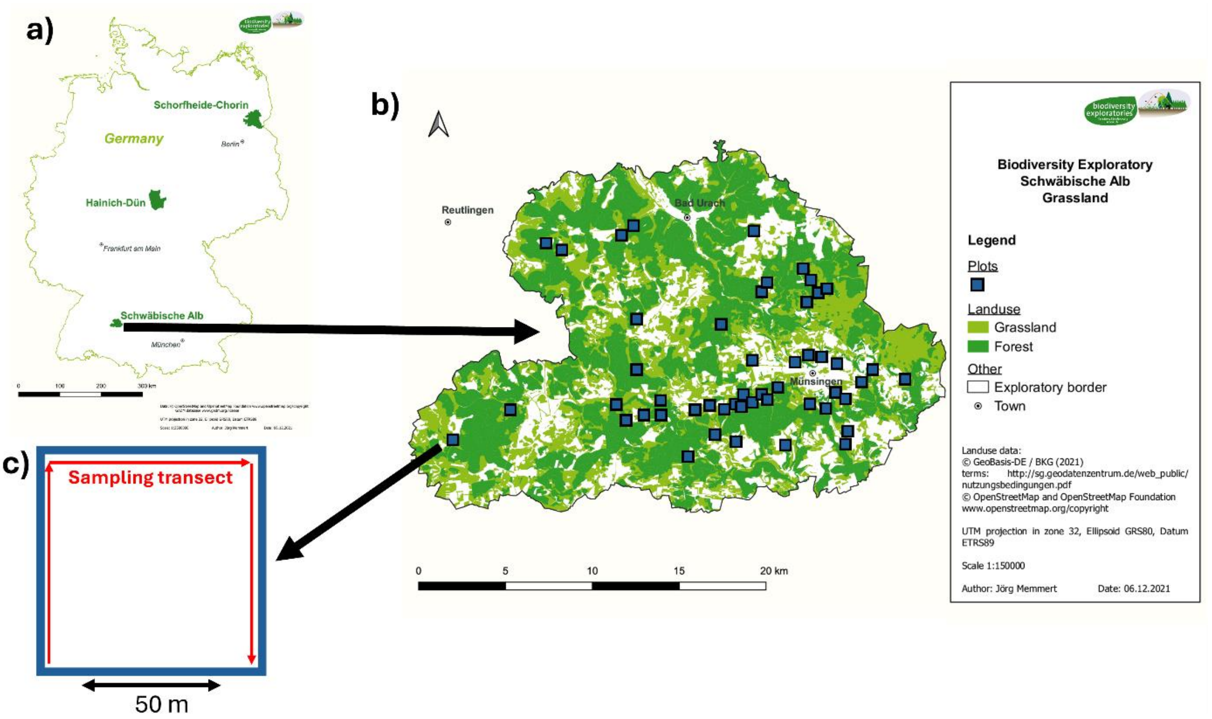

Supplementary Figure 9 ***Map of the Biodiversity Exploratories****, modified after Jörg Memmert. The three study regions are distributed across Germany (a), with 50 plots per study region (b; here the region Schwäbische Alb is shown). Each plot has an area of 50x50 m and was sampled using sweep netting along transects of 150 m in total (see inset graph and methods in the main manuscript).*

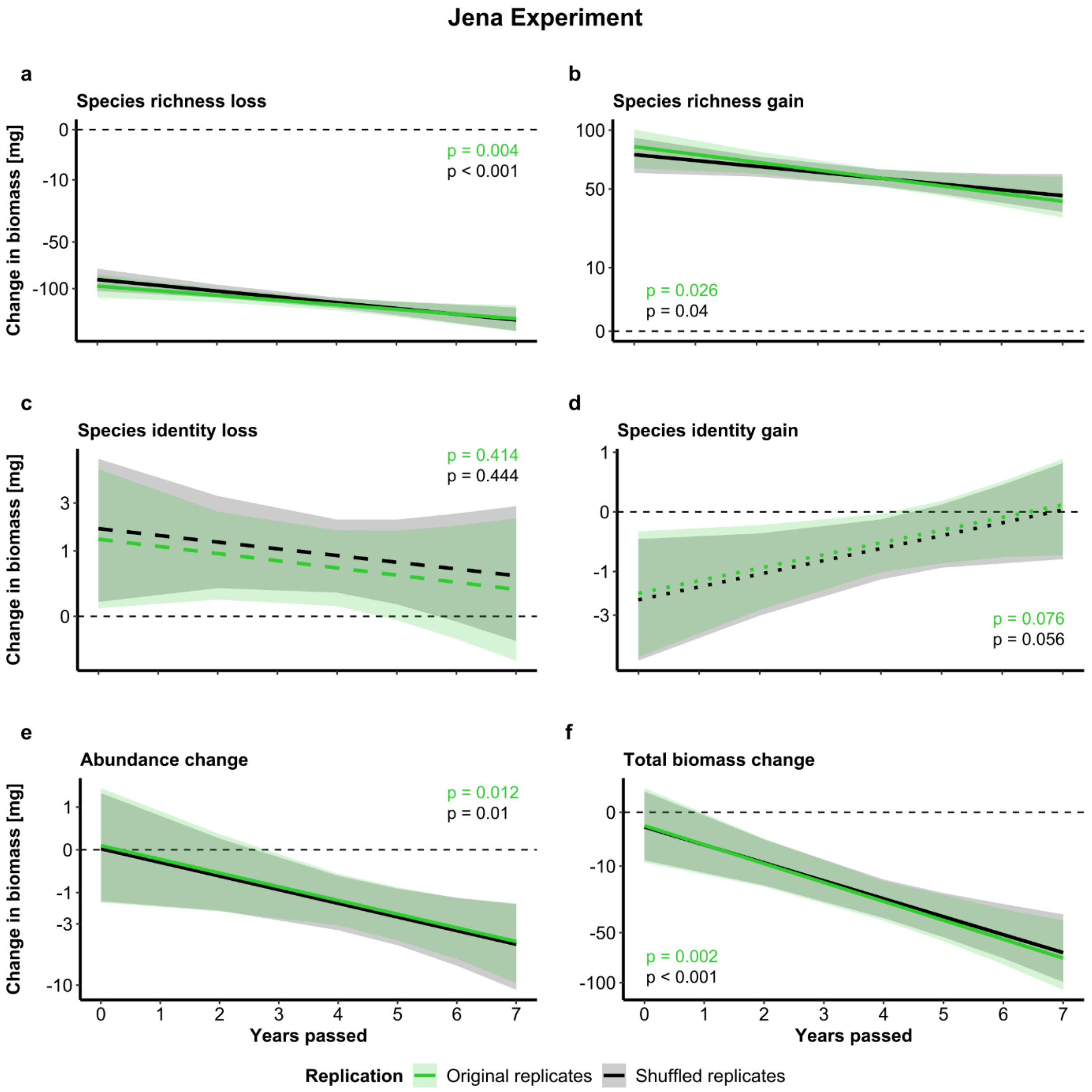

Supplementary Figure 10 ***Comparison of modelled temporal trends of partitioned biomass change (restricted moving average) between the original replicates (black) and the shuffled replicates (green) per replicate (n = 160) in the Jena Experiment.*** *All panels are based on median point estimates and predictions from 1,000 linear mixed-effects models drawing from shuffled data subsets, avoiding the reuse of sampling events in multiple pairwise comparisons. Temporal biomass change was associated with a) species richness loss (assuming average biomass of lost species relative to their respective communities), b) species richness gain (assuming average biomass of gained species relative to their respective communities), c) species identity loss (deviation of observed biomass change associated with lost species from the expected biomass change associated with species richness loss), d) species identity gain (deviation of observed biomass change associated with gained species from the expected biomass change associated with species richness gain), and e) abundance change of persisting species. f) Total biomass change without partitioning. Solid lines indicate two-sided p < 0.05 (significant), dotted lines indicate p < 0.1 (marginally significant), dashed lines indicate non-significant relationships; exact p-values are provided in the panels.*

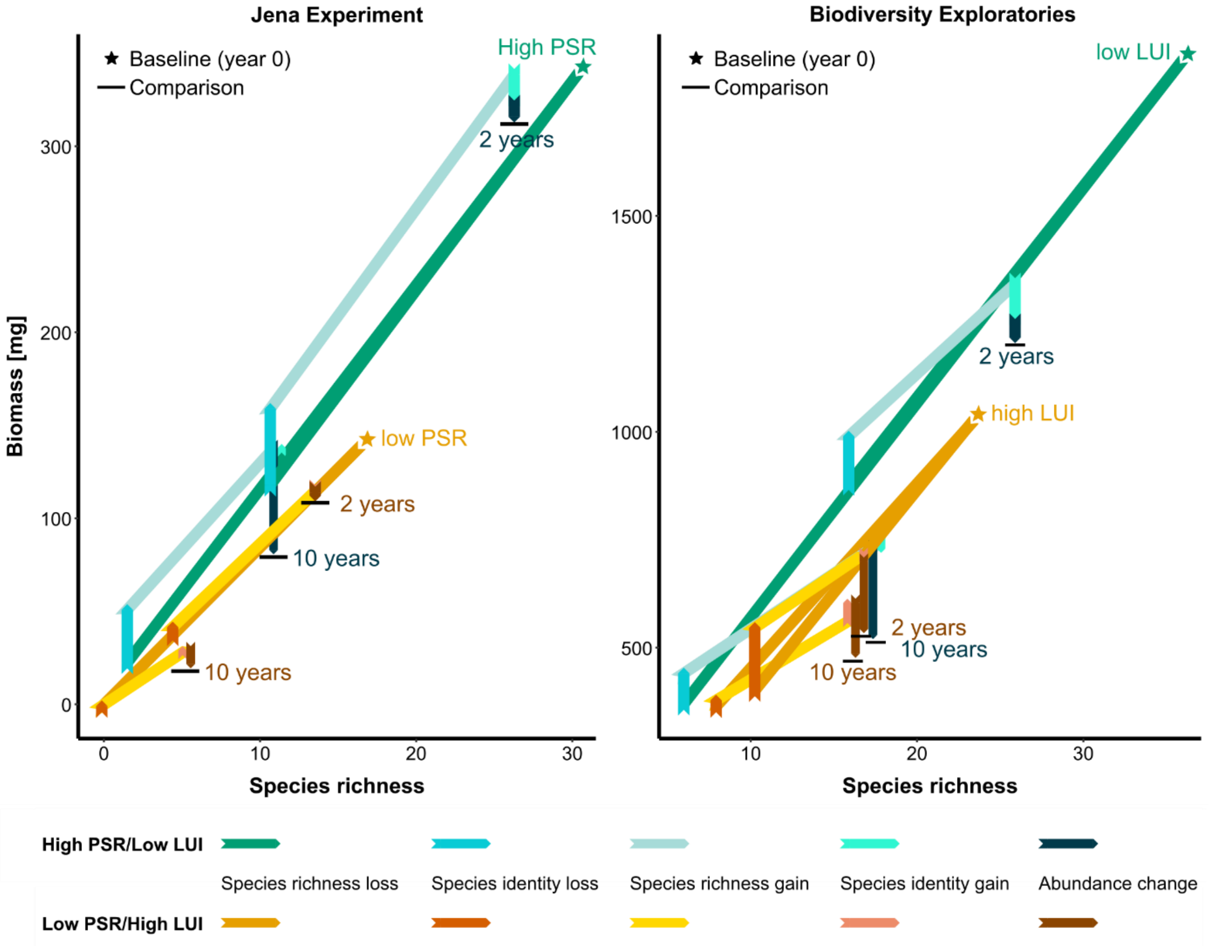

Supplementary Figure 11 ***Modelled change of arthropod biomass (y) and species richness (x) per replicate per plot after 2 and 10 years (fixed baseline) in dependence of plant species richness (PSR) and land-use intensity (LUI).*** *Starting at the average community biomass and species richness value in the first year (Baseline), the community assembly components of biomass change are displayed as vectors (arrows) in the order of 1) species richness loss, 2) species identity loss, 3) species richness gain, 4) species identity gain, and 5) abundance change of persisting species (see Fig 2 for detailed explanations), reaching the predicted comparison community values of absolute biomass and species richness after the respective timespans of 2 and 10 years. Most arthropod biomass change is associated with species richness change, but the vertical vectors of species identity and abundance change show that species richness alone does not explain all biomass change (see Supplementary Note 1). Vectors are based on median predictions from 1,000 linear mixed-effects models (see methods and Tables S20, S21). Vectors for high PSR (60 plant species) and low LUI (0.5) plots are colored in green/blue, vectors for low PSR (monoculture) and high LUI (3.5) plots are colored in beige/brown (see legend).*

Supplementary References

1. Bannar-Martin, K. H. *et al.* Integrating community assembly and biodiversity to better understand ecosystem function: the Community Assembly and the Functioning of Ecosystems (CAFE) approach. *Ecology Letters* **21**, 167–180 (2018).
2. Seibold, S. *et al.* Arthropod decline in grasslands and forests is associated with landscape-level drivers. *Nature* **574**, 671–674 (2019).
3. Bourrat, P. *et al.* What is the price of using the Price equation in ecology? *Oikos* **2023**, e10024 (2023).
4. Müller, J. *et al.* Weather explains the decline and rise of insect biomass over 34 years. *Nature* **628**, 349–354 (2024).
5. Pigot, A. L. *et al.* Macroecological rules predict how biomass scales with species richness in nature. *Science* **387**, 1272–1276 (2025).
6. Gossner, M. M. *et al.* Land-use intensification causes multitrophic homogenization of grassland communities. *Nature* **540**, 266–269 (2016).
7. Bröcher, M., Meyer, S. T., Leher, A. G. & Ebeling, A. Ecological traits for 1374 arthropod species collected in a German grassland. *Ecology* **106**, e70077 (2025).
8. Gossner, M. M. *et al.* A summary of eight traits of Coleoptera, Hemiptera, Orthoptera and Araneae, occurring in grasslands in Germany. *Sci Data* **2**, 150013 (2015).
